# Supplementary material for: Non-CG methylation and multiple histone profiles associate child abuse with immune and small GTPase dysregulation
Source: Nat Commun. 2021 Feb 18;12:1132. doi: 10.1038/s41467-021-21365-3 (PMC7892573; doi:10.1038/s41467-021-21365-3)
Supplement: Supplementary file 1 — Supplementary Information [file 41467_2021_21365_MOESM1_ESM.pdf]

1. Supplementary Figures legends

Supplementary Figure 1. Quality controls for chromatin immunoprecipitation sequencing (ChIP-Seq). (a) ChIP-Seq libraries were prepared and sequenced by expert technicians at the Genome Québec Innovation Center, in the framework of the International Human Epigenomics Consortium (IHEC). We compared psychiatrically healthy controls (C, n=4 pools; see also Supplementary Table 3 below) and subjects with a history of early-life adversity (ELA, n=7 pools), and analyzed 4 'broad' (H3K9me3, H3K27me3, H3K36me3, H3K4me1) and 2 'narrow' (H3K4me3, H3K27ac) marks, for which we aimed at sequencing roughly 60 and 30 million reads per library, respectively, as per the IHEC consortium's guidelines. A two-way ANOVA indicated that there was no difference among C and ELA groups in terms of sequencing depths [$F(1,63)=0.52$; $p=0.47$]. Values are mean±sem. **(b)** Quality controls analyses showed that samples for narrow marks showed greater than 0.8 and 1.05 Relative Strand Cross and Normalized Strand Cross correlations, respectively, thereby meeting ENCODE consensus thresholds for quality control[1].

Supplementary Figure 2. Comparison of amygdalar histone profiles for individual marks with datasets from inferior temporal lobe (Inf Temp), anterior caudate (Ant Caud), and peripheral blood mononuclear cells (Blood). Data were downloaded from the Roadmap Epigenomics Consortium (ncbi.nlm.nih.gov/geo/roadmap/epigenomics/) and compared to each C and ELA amygdalar sample from the present study, using deepTools Pearson correlations and unsupervised clustering[2], for each of the 6 histone marks: **(a)** H3K4me1; **(b)** H3K27ac; **(c)** H3K4me3; **(d)** H3K36me3; **(e)** H3K9me3; **(f)** H3K27me3. Accession numbers: 1) for inferior temporal lobe: datasets GSM772995 (H3K27ac), GSM772993 (H3K27me3), GSM772982 (H3K36me3), GSM772992 (H3K4me1), GSM772996 (H3K4me3), GSM772994 (H3K9me3); 2) for anterior caudate: datasets GSM772832 (H3K27ac), GSM772827 (H3K27me3), GSM772828 (H3K36me3), GSM772830 (H3K4me1), GSM772829 (H3K4me3), GSM772831 (H3K9me3); 3) for blood mononuclear cells: datasets GSM1127145 (H3K27ac), GSM1127130 (H3K27me3), GSM1127131 (H3K36me3), GSM1127143 (H3K4me1), GSM1127126 (H3K4me3), GSM1127133 (H3K9me3). Of note, H3K4me1, H3K27ac and H3K27me3 better discriminated between tissue types than the 3 other marks.

Supplementary Figure 3. Characterization of amygdalar chromatin states defined using ChromHMM, and comparison with external datasets from human brain hippocampus and gastric tissues. (a) A snapshot of the ChromHMM 10-state model that was generated using amygdalar data for 6 histone marks is shown. **(b)** The graph depicts the number of genomic regions identified for each chromatin state; each region is defined as continuous genomic bins showing a similar combination of individual histone marks in the consensus

ChromHMM model (see Methods). **(c)** Distribution of genomic size of regions identified for each chromatin state (with the number of regions identified for each state indicated in panel b). Box plots show median and interquartile range, with whiskers representing minimum and maximum values. **(d)** Methylation levels in the CAC context (y-axis) were plotted against the region size (x-axis) for the 3 Promoter states: Active Promoter (Act Prom, upper panel), Weak Promoter (Wk Prom, middle panel), and Flanking Promoter (Flk Prom, lower panel). **(e-f)** Comparison of the amygdalar ChromHMM 10-state consensus model generated here using amygdalar tissue with similar chromHMM models generated by the NIH Roadmap. Brain hippocampus and gastric tissue datasets were downloaded from the Roadmap Epigenomics Project portal (Epigenome ID E071 and E094 respectively, for the core 15-state model, egg2.wustl.edu/roadmap/web_portal/chr_state_learning.html) and compared using Bedtool's Jaccard coefficient to our consensus map (generated using ChromHMM, see *Methods*). Note that our amygdala chromatin model more strongly correlates with that of the brain hippocampus than with gastric tissue, as expected.

Supplementary Figure 4. Relationship between chromHMM chromatin state and gene expression levels. For each gene (+/- 3kb), the percentage of its genomic span covered by each chromatin state was computed. Values were then ordered by gene expression level and averaged over bins of 500 genes (values depicted are mean ± sem). Here are shown the average percentage coverage of genes by **(a)** Weak transcription (Wk Trans) and Strong transcription (Str Trans), **(b)** Genic enhancer (Gen Enh) and Enhancer (Enh), **(c)** Active promoter (Act Prom), Weak promoter (Wk Prom) and Flanking promoter (Flk Prom) states. These chromatin states exhibited expected correlations with gene expression: (i) the Wk Trans state was more frequently observed in gene bodies of lowly expressed genes compared with the Str Trans state, (ii) enhancer states were enriched in genes with strong expression and (iii) the Wk Prom state was more frequently observed in lowly expressed genes compared with the Act Prom state. Values are mean±sem.

Supplementary Figure 5. Quality controls for whole-genome bisulfite sequencing libraries (WGBS). To compare DNA methylation patterns among psychiatrically healthy individuals (controls, C) and subjects with a history of severe child abuse (early-life adversity, ELA), WGBS libraries were prepared. **(a)** Bisulfite conversion efficiencies were measured for each DNA sample using spiked-in unmethylated lambda DNA, and were similar between groups: C: 99.3±0.07%, ELA: 99.2±0.06% (two-sided Student t-test: t=0.63, p=0.53). **(b)** In addition, over-conversion (i.e. methylated cytosines converted to uraciles during bisulfite conversion) was determined experimentally using spiked-in fully methylated pUC19 DNA, and similar values were observed between groups: C: 5.6±0.10%, ELA: 5.7±0.05% (two-sided

Student t-test: t=0.19, p=0.85). **(c)** Each WGBS library was then sequenced on 1 lane of a HiSeq 2000 (100 base pair, paired-end sequencing) at the Génome Québec Innovation Center, yielding similar sequencing depth across groups: C: 164±3 million reads, ELA: 163±3 million reads (Student t-test: t=0.15, p=0.88). **(d)** During the processing of raw sequencing data, duplicates were removed from downstream analysis, and results indicated similar diversity (Student t-test: t=1.18, p=0.25) among libraries from the C (C:22.5±1.2%) and ELA (C:20.8±0.9%) groups. **(e)** The graphs depicts the number of CG sites that met distinct average coverages among the 38 WGBS libraries. For the characterization of genome-wide abundance and distribution of CG and CAC methylation levels (Fig.2 and Supplementary Fig.6-9), we focused on cytosines with a coverage >= 5. Abbreviations: cov., coverage. Values are mean±sem.

Supplementary Figure 6. Distribution of non-CG DNA methylation in the human brain lateral amygdala. (a) The graph depicts the distribution of genome-wide average non-CG methylation levels that were observed in the human brain lateral amygdala in healthy controls (C, n=17 subjects), and subjects with a history of child abuse (early-life adversity, ELA, n=21 subjects). The ordering of these 12 non-CG contexts at bulk tissue level is identical to one described by Mo et al[3] in the mouse at cell-type specific level for 3 neuronal populations: glutamatergic neurons (Exc), parvalbumin-expressing (PV) and vasoactive intestinal peptide (VIP)-expressing inhibitory neurons. Values are mean±sem, shown here for the whole cohort (combined C and ELA groups, n=38 subjects). **(b)** While most cytosines in non-CG contexts were unmethylated, a minority of these positions nevertheless showed methylation levels between 5 and 25%, whatever the 3-letter context considered, with a peak between 15 to 20%. These results indicate that while different numbers of cytosines might be methylated across various non-CG contexts, the abundance of DNA methylation (i.e., the proportion of cells affected) at those sites seems relatively homogeneous. Box plots show median and interquartile range, with whiskers representing minimum and maximum values

Supplementary Figure 7. Distribution of CG and CAC DNA methylation among distinct genomic features and chromosomes. (a) CAC and CG methylation levels were computed across distinct genomic features defined using the region_analysis package[4]. In the CG context, DNA methylation levels strongly varied as a function of the genomic feature (two-sided 2-way ANOVA, [F(7,252)=953; p<0.0001]), while there was no significant difference [F(1,36)=0.50; p=0.48] among psychiatrically healthy individuals (C, n=17 subjects) and subjects with a history of early-life adversity (ELA, n=21 subjects). Post-hoc comparisons confirmed that, as expected, lowest CG methylation levels were observed in promoter regions, in particular within a 250-base pair distance from the TSS (ProximalPromoter), where

methylation levels were significantly lower than in any other gene feature (p<0.0001). In the CAC context, similar significant and non-significant effects were found for genomic features [F(7,252)=917; p<0.0001] and for clinical grouping [F(1,36)=0.03; p=0.85], respectively. In contrast with the CG context, lowest CAC methylation levels were observed in pericentromeric regions (defined by region_analysis as regions located between the boundary of a centromere and the closest gene minus 10kbp of that gene's regulatory region[4]), which showed strongly significant differences with all other features (p<0.0001). Values are mean±sem. **(b)** CAC and CG methylation levels were computed in each chromosome across the whole cohort. As expected, DNA methylation levels were much higher in the CG than in the CAC context (2-way ANOVA; context effect: [F(1,1850)=1238951; p<0.0001]), and methylation abundance strongly varied among chromosomes in each context (chromosome effect: [F(24,1850)=2673; p<0.0001]). As expected also, methylation was extremely low in the mitochondrial genome in both the CG and CAC contexts. Values are mean±sem, shown here for the whole cohort (combined C and ELA groups, n=38 subjects).

Supplementary Figure 8. Correlations between the expression of genes and DNA methylation levels in their sense or antisense strands, in the CG and CAC contexts. The 1000 most highly (top1000) and 1000 most lowly expressed genes were identified using RNA-Sequencing data, and compared for abundance of DNA methylation in: **(a,d)** both DNA strands; **(b,e)** the strand where genes are located (sense strand), and **(c,f)** the strand antisense to the one where genes are located. Negative correlations between DNA methylation and gene expression were observed in all cases: **(a)** [F(1,74)=736.1; p<2E-16] **(b)** [F(1,74)=742.8; p<2E-16] **(c)** [F(1,74)=615.1; p<2E-16] **(d)** [F(1,74)=145.3; p<2E-16] **(e)** [F(1,74)=119.2; p<2E-16] **(f)** [F(1,74)=142.6; p<2E-16] (2-way repeated measures ANOVA, main effects of gene category, top1000 versus bottom1000 averaged over 100 bins). Results therefore indicate that gene expression is predicted to the same extent by mCAC on either strand, at least for the coverage achieved in this study.

Supplementary Figure 9. Annotation of DNA methylation features in the human brain lateral amygdala. (a) Unmethylated (UMR) and lowly methylated (LMR) genomic regions were identified using the methylseekR algorithm, as described in[5]. No partially methylated domains (PMDs) were identified, similar to previous studies on mouse retina[6], human embryonic stem cells and neural progenitor cells[7]. **(b-c)** LMR (n=115254) and UMR (n=21267) showed methylation levels in the CG context (b) and size (c) consistent with previous reports[5], while UMR (mean ± sem = 2516 ± 41 bp) were generally larger than LMR (738 ± 107 bp). Dashed and dotted lines represent medians and quartiles, respectively. **(d)** Among all CpG islands in the human genome (RefSeq reference, n= 28691), a vast majority (n=19193;

n=66.9%) intersected with CpG-dense UMR**,** while a small minority corresponded to LMR (n=2743; 9.6%), as described previously for other tissue types[7]. **(e)** Methylation in the CG (mCG) and CAC (mCAC) contexts were computed across RefSeq genomic features, UMR and LMR, and found to strongly differ, as expected. While mCAC levels are much lower than mCG levels, a similar pattern of variation is observed across the 2 cytosine contexts. Values are mean±sem, shown here for the whole cohort (combined C and ELA groups, n=38 subjects). **(f)** mCG (left panel) and mCAC (right panel) levels were computed in each intersection among RefSeq genomic features, LMR, or UMRn and chromatin states (identified using ChromHMM, see *Methods*), across our entire cohort. Of note, while mCG abundance in LMRs remained constant between 25 and 35% across all chromatin states, mCAC was more variable, and notably lower in Polycomb Repressed (PcR), heterochromatin (Heterochr) and Strong Transcription (Str Trans) states. Also, mCAC was more abundant in Wk Trans than in Str Trans within genes, consistent with the fact that Wk Trans was more abundant in lowly expressed genes (see Supplementary Fig.9a), and previous reports[8].

Supplementary Figure 10. Histone profiles at lowly methylated regions (LMR) and unmethylated regions (UMR). (a) Enrichment of chromatin states at and around positions of UMR (left panel) and LMR (right panel) were evaluated using chromHMM's NeighborhoodEnrichment function. As expected, UMRs co-located with Weak and Active Promoters (Wk Prom, Act Prom), and were flanked by the Flanking Promoter (Flk Prom) state. In contrast, LMRs were consistently surrounded by Str Enh and Enh states, which spanned 2 and 1 Kb around LMR positions, respectively, consistent with their role as distinct regulatory element[7]. **(b-g)** Average enrichments of ChIP-seq reads over input are shown for each histone mark within LMR and UMR, as well as in flanking up- and down-stream regions (+/- 1 Mb). Patterns are consistent with those reported by Stadler et al[7] for 2 marks (H3K4me1, H3K4me3), and provide new information related to the other 4 marks: i) LMR and UMR associated with increased levels of H3K4me1, H3K4me3 and H3K27ac, and these effects were more pronounced for UMR than for LMR, consistent with the fact that CpG islands showed a stronger overlap with UMR than with LMR (FigS7d); ii) The other 3 marks (H3K36me3, H3K9me3, H3K27me3) were depleted within UMR and LMR; iii) interestingly, while LMR regions showed uniform enrichment or depletion throughout their whole genomic span, more complex patterns were observed for UMR. Their enrichment for H3K4me1 showed a biphasic pattern, and preferentially affected their 5' and 3' shores, very similar to results obtained by Stadler et al in mouse embryonic stem cells; further, this biphasic enrichment was also present for H3K4me3 and H3K27ac, albeit to a lesser extent, and was mirrored by a biphasic H3K9me3 depletion in UMR shores.

Supplementary Figure 11. Distributions of histone reads across gene bodies and differential sites (DS). For each histone mark, the figure depicts the distribution of reads (average enrichment of ChIP-seq reads over input) across all gene bodies (All Genes). In addition, and as a control, we also analyzed their distribution at genomic sites where Up- and Down-DS were identified between control (C, n=4 pools) and early-life adversity (ELA, n=7 pools) groups. Values are mean±sem.

Supplementary Figure 12. Identification of genomic features where histone differential sites (DS) were localized. (a) Localization of DS (identified using diffRep, see *Methods*) among distinct genomic features (defined using region_analysis[4]), for each histone mark. The observed distributions were significantly different across histone marks (df=25, χ^2 =1244, p<0.001). In addition, comparisons between observed and expected (genome-wide distribution of reads among genomic features in the 2 C and ELA groups combined) distributions showed that, for each type of histone modification, ELA-associated DS were non-randomly located in specific genomic features: H3K27ac (df=1, χ^2 =81.2, p<0.001); H3K27me3 (df=1, χ^2 =42.3, p<0.001); H3K36me3 (df=1, χ^2 =138, p<0.001); H3K4me1 (df=1, χ^2 =287, p<0.001); H3K4me3 (df=1, χ^2 =90.9, p<0.001); H3K9me3 (df=1, χ^2 =22.6, p<0.001). **(b)** Analysis of the directionality of DS showed that ELA more frequently associated with decreases (Down-DS) than increases (Up-DS) in read density, as found for 4 marks: H3K4me1 (df=1, χ^2 =231, p<0.001); H3K4me3 (df=1, χ^2 =73, p<0.001); H3K36me3 (df=1, χ^2 =345, p<0.001); H3K27me3 (df=1, χ^2 =228, p<0.001). DS were equally distributed among Up- and Down-DS for the 2 remaining marks: H3K27ac (df=1, χ^2 =0.13, p=0.19); H3K9me3 (df=1, χ^2 =1.7, p=0.19). **(c)** Integrin signaling is enriched across multiple histone changes and state transitions. GREAT pathway analysis using MsigDB showed recurrent enrichment of the integrin signaling pathway across six types of state transitions, as well as for H3K27ac down-DS (differential sites). Each analysis passed hypergeometric and binomial testing (fold change ≥ 1.5 and Q ≤ 0.1 for both tests). Negative logarithmic P-value is shown for the binomial test**.** Chromatin states: Str-Trans, strong transcription; Wk-Trans, weak transcription; Str-Enh, strong enhancer; Enh, enhancer.

Supplementary Figure 13. Comparison of main metrics for CG and CAC differentially methylated regions (DMR). (a-d) 840 CAC- and 795 CG-DMRs were identified using BSmooth (see *Methods*), and metrics are compared here for the 2 categories of DMRs. Box plots show median and interquartile range, with whiskers representing minimum and maximum values. **(a)** Compared to CG-DMRs, CAC-DMRs were composed of slightly fewer cytosines (CG: 7.63±0.11; CAC: 7.14±0.08; two-sided Student t-test: [t(1,1633)=4.12, p=4.0E-05]), and **(b)** were smaller (CG: 321±7 bp; CAC: 245±4 bp; two-sided Student t-test: [t(1,1633)=10.54, p<1E-10]). The amplitude of methylation changes observed in subjects from the ELA group

was smaller in the CAC than in the CG context, as shown by **(c)** smaller % changes in methylation levels (CG: 7.75±0.05%; CAC: 4.6±0.03%; two-sided Student t-test: [t(1,1633)=59.0], p<1E-10), and **(d)** smaller areaStat values (a metric measuring the statistical strength of methylation changes among cytosines composing each DMR[9]; CG: 32.3±0.5%; CAC: 28.9±0.4%; two-sided Student t-test: [t(1,1633)=6.1], p=1.6E-09). **(e)** Visualization of all CG- and CAC-DMRs across the human genome using chromPlot. There was absolutely no intersection between the 2 types of DMRs.

Supplementary Figure 14. Comparison of histone mark profiles at CG and CAC DMRs. (a-f) Average enrichments of ChIP-seq reads over input are shown for each histone mark around each type of DMRs (CG, green n=795; CAC, orange, n=840): **(a)** H3K4me1, **(b)** H3K9me3, **(c)** H3K4me3, **(d)** H3K36me3, **(e)** H3K27ac, and **(f)** H3K27me3. Histone reads enrichment significantly varied within DMRs and flanking regions (+/- 2 kilobases, kb) for the 6 marks (2-way repeated measures ANOVA, main effects of cytosine position, x-axis; p<0.0001). Also, significant differences in histone reads density (y-axis) among the 2 cytosine contexts were observed for 4 marks (H3K27ac, H3K4me3, H3K27me3, H3K4me1; p<0.05), but not for H3K9me3 (p=0.08) or H3K36me3 (p=0.57). Importantly, significant interactions between histone reads density and cytosine position within DMRs were observed for all marks (p<0.0001). In particular, post-hoc comparisons confirmed that, compared with their flanking regions, CAC-DMRs were significantly enriched for H3K36me3 and depleted in H3K9me3; in contrast, CG-DMRs were characterized by significant enrichments for H3K4me1, H3K4me3, and H3K27ac, and a depletion in H3K36me3 (p<0.0001 for each post-hoc comparison). Values are mean±sem across the n=11 pools. **(g)** Enrichment of each chromatin state over CG and CAC differentially methylated regions (DMRs). The figure depicts the enrichment of each chromatin state (as identified using ChromHMM and the combination of all 6 histone marks, see main text) among genomic regions corresponding to CG- and CAC-DMRs, compared to their relative abundance in the overall human genome. CG-DMRs shown in blue, CAC-DMRs shown in red. Chromatin states: Act-Prom, active promoter; Wk-Prom, weak promoter; Flk-Prom, flanking promoter; Str-Trans, strong transcription; Wk-Trans, weak transcription; Str-Enh, strong enhancer; Enh, enhancer; PcR, polycomb repressed; Hetero, heterochromatin.

Supplementary Figure 15. RNA-Sequencing quality controls. Total RNAs extracted from lateral amygdala tissue (controls, C, n=17; early-life adversity, ELA, n=21) were used for the preparation of RNA-Sequencing libraries and processed in parallel. **(a)** RNA integrity values (RIN) were not significantly different across RNA samples extracted from C (n=17) and ELA (n=21) subjects (Mann-Whitney U=171; p=0.83). **(b)** The number of reads sequenced in each library was similar across C and ELA groups (two-sided Student t-test: t=0.72; p=0.48). **(c-d)**

Similarly, there was no significant difference in percentages of duplicates (two-sided Student t-test: $t=1.25$; $p=0.22$) nor in alignment rate (two-sided Student t-test: $t=0.51$; $p=0.62$) between the 2 groups. Values are mean±sem.

Supplementary Figure 16. RNA-Sequencing results. **(a)** Very similar results (linear regression: $r=0.82$, $p< 2.2E-16$) were obtained using 2 distinct bio-informatic pipelines for the analysis of RNA-Sequencing data. Raw reads were aligned & counted using either HTSeq-count or Kallisto, followed by the analysis of differential expression between C and ELA groups using DESeq2. FC, Fold Change. **(b)** Genome-wide gene expression changes occurring in the lateral amygdala (this study) and the anterior cingulate cortex (ACC, our previous work[14]) as a function of ELA were compared using the RRHO2 algorithm[15]. Results uncovered strong patterns of common dysregulation, whereby 2 large groups of genes show similar down- (p-adj=10^{-487}, Benjamini-Yekutieli correction for multiple testing) or up-regulation (10^{-388}) or in both regions in ELA subjects (see lower-left quadrant, UP/UP, and upper-right quadrant, DOWN/DOWN). **(c)** The 2 gene lists obtained from RRHO2 were then used for Gene Ontology enrichment analysis (FDR<0.1). Results showed that a large number of GO terms previously identified during multi-epigenetic analysis of the single amygdala dataset (see panel e below) also emerged from the combined analysis of transcriptomes from both regions. These results suggest that at least part of the transcriptional changes observed in the amygdala also affect another brain region, the ACC, that significantly contributes to mood regulation and depression pathophysiology. **(d-e)** Gene Set Enrichment Analysis (GSEA) of RNA-Sequencing data in the lateral amygdala. Genes were ranked based on their log_2 fold changes ('Ranked list metric', from the differential gene expression analysis between C and ELA groups, represented in grey in the lower portion of each panel); genes with highest positive fold changes (in red, upregulated in the ELA group) were at the extreme left of the distribution, and those with lowest negative fold changes (in blue, downregulated in the ELA group) were at the extreme right of the distribution. A running enrichment score (green line, upper portion of each panel) was computed for gene sets from the C2 MSigDB curated molecular signatures database, and used to identify enriched gene sets[16]. Depicted are the 2 single gene sets that achieved the highest normalized enrichment scores (with the middle portion of each panel showing vertical black lines where members of the gene set appear in the ranked list of genes). **(d)** A collection of genes related to oligodendrocytes and myelin physiology (left panel), which we recently found downregulated in the ACC of subjects with a history of ELA[14], showed an opposite upregulation in the lateral amygdala (GSEA normalized enrichment score, NES=2.91; family-wise error rate, FWER p-value<0.05), suggesting that opposed transcriptional adaptations might occur as a function of ELA between cortical and subcortical structures in this glial population. The right panel depicts log2 FC in both the lateral amygdala and ACC for this

myelin genes collection. **(e)** These finding were reinforced by the second-best gene set, which was significantly enriched for upregulated genes in our amygdala data (GSEA normalized enrichment score, NES=2.75; family-wise error rate, FWER p-value<0.05). The later gene collection was previously associated with depression in the middle temporal gyrus[17], and found enriched in myelin-related genes, suggesting that similar stress-related transcriptomic changes may affect oligodendrocytes among distinct portions of the temporal lobe. **(f)** Identification of Gene ontology (GO) processes most consistently affected by ELA, as identified by the combined analysis of individual histone marks, chromatin states, DNA methylation, and gene expression (see main text).

Supplementary Figure 17. Deconvolution of WGBS and RNA-Sequencing data. (a) Consistent with the cortical nature of the lateral nucleus of the amygdala and with estimates from neuroanatomical studies[10,11], RNA-Seq deconvolution using BSEQ-sc Cibersort[12] indicated that the neuronal population in our samples was composed of 80% excitatory and 20% inhibitory neurons. Importantly, we found no differences in cell-type composition between C (n=17 subjects) and ELA (n=21 subjects) groups (2-way ANOVA; cell-type effect p<0.0001; group effect, p=0.61), whether at the level of 5 major cell types (excitatory and inhibitory neurons, microglia, astrocytes, oligodendroglia, endothelial cells) **(b),** or when considering the full spectrum of 26 cell-type clusters that were identified **(c)**. We then deconvoluted WGBS data using the same Cibersort algorithm and, to our knowledge, the only available dataset for single-cell methylomes in the human brain[13]. Despite the fact that only non-CG methylation levels, but not CG methylation, are available from the later study, we nevertheless observed a significant correlation between the estimated proportion of excitatory neurons in this second approach, and the proportion of the most abundant population of excitatory neurons (Ex_5_L5) observed during deconvolution of RNA-Seq data **(d)**, indicating some convergence between the 2 deconvolutions. **(e)** Importantly, consistent with RNA-Sequencing data, no significant difference in cell-type proportions were identified among C and ELA groups in WGBS deconvolution (2-way ANOVA; cell-type effect p<0.0001; group effect, p=0.11). Panel a, values are mean±sem; panels b, c and d, box plots show median and interquartile range, with whiskers representing minimum and maximum values.

Supplementary References

1. Landt, S. G. *et al.* ChIP-seq guidelines and practices of the ENCODE and modENCODE consortia. *Genome Res* 22, 1813–31 (2012).

2. Bernstein, B. E. *et al.* The NIH Roadmap Epigenomics Mapping Consortium. *Nat Biotechnol* 28, 1045–8 (2010).

3. Mo, A. *et al.* Epigenomic Signatures of Neuronal Diversity in the Mammalian Brain.

Neuron 86, 1369–1384 (2015).
4. Shen, L. *et al.* diffReps: detecting differential chromatin modification sites from ChIP-seq data with biological replicates. *PLoS One* 8, e65598 (2013).
5. Burger, L., Gaidatzis, D., Schubeler, D. & Stadler, M. B. Identification of active regulatory regions from DNA methylation data. *Nucleic Acids Res* 41, e155 (2013).
6. Dvoriantchikova, G., Seemungal, R. J. & Ivanov, D. The epigenetic basis for the impaired ability of adult murine retinal pigment epithelium cells to regenerate retinal tissue. *Sci Rep* 9, 3860 (2019).
7. Stadler, M. B. *et al.* DNA-binding factors shape the mouse methylome at distal regulatory regions. *Nature* 480, 490–5 (2011).
8. Stroud, H. *et al.* Early-Life Gene Expression in Neurons Modulates Lasting Epigenetic States. *Cell* 171, 1151-1164 e16 (2017).
9. Hansen, K. D., Langmead, B. & Irizarry, R. A. BSmooth: from whole genome bisulfite sequencing reads to differentially methylated regions. *Genome Biol* 13, R83 (2012).
10. Prager, E. M., Bergstrom, H. C., Wynn, G. H. & Braga, M. F. The basolateral amygdala gamma-aminobutyric acidergic system in health and disease. *J Neurosci Res* 94, 548–67 (2016).
11. Sah, P., Faber, E. S., Lopez De Armentia, M. & Power, J. The amygdaloid complex: anatomy and physiology. *Physiol Rev* 83, 803–34 (2003).
12. Newman, A. M. *et al.* Robust enumeration of cell subsets from tissue expression profiles. *Nat Methods* 12, 453–7 (2015).
13. Luo, C. *et al.* Single-cell methylomes identify neuronal subtypes and regulatory elements in mammalian cortex. *Science* 357, 600–604 (2017).
14. Lutz, P. E. *et al.* Association of a History of Child Abuse With Impaired Myelination in the Anterior Cingulate Cortex: Convergent Epigenetic, Transcriptional, and Morphological Evidence. *Am J Psychiatry* 174, 1185–1194 (2017).
15. Plaisier, S. B., Taschereau, R., Wong, J. A. & Graeber, T. G. Rank-rank hypergeometric overlap: identification of statistically significant overlap between gene-expression signatures. *Nucleic Acids Res* 38, e169 (2010).
16. Subramanian, A. *et al.* Gene set enrichment analysis: a knowledge-based approach for interpreting genome-wide expression profiles. *Proc Natl Acad Sci U S A* 102, 15545–50 (2005).
17. Aston, C., Jiang, L. & Sokolov, B. P. Transcriptional profiling reveals evidence for signaling and oligodendroglial abnormalities in the temporal cortex from patients with major depressive disorder. *Molecular Psychiatry* 10, 309–22 (2005).

2. Supplementary Figures

Supplementary Figure 1

a

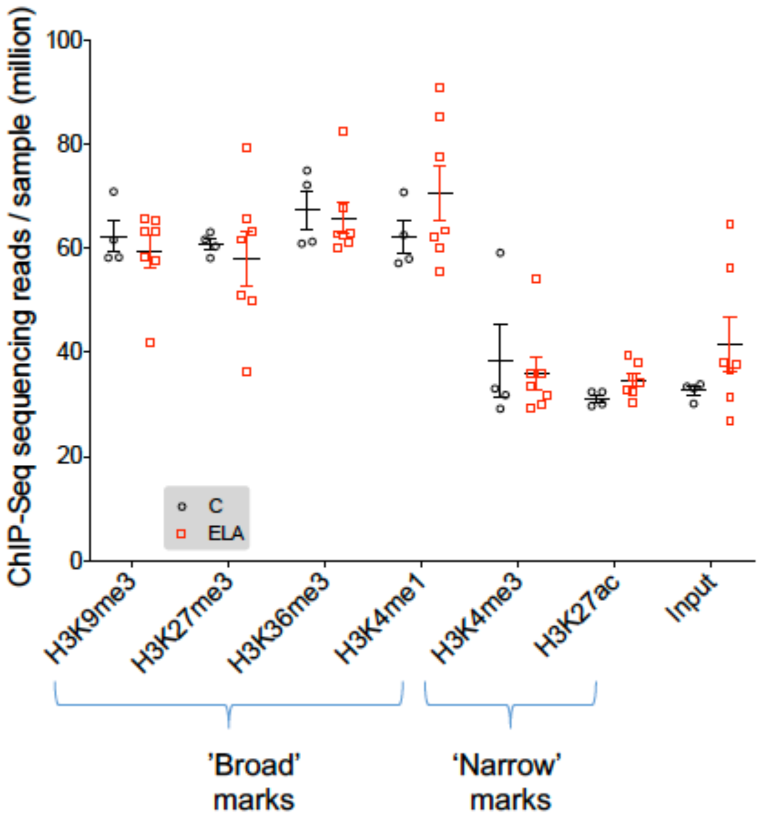

b

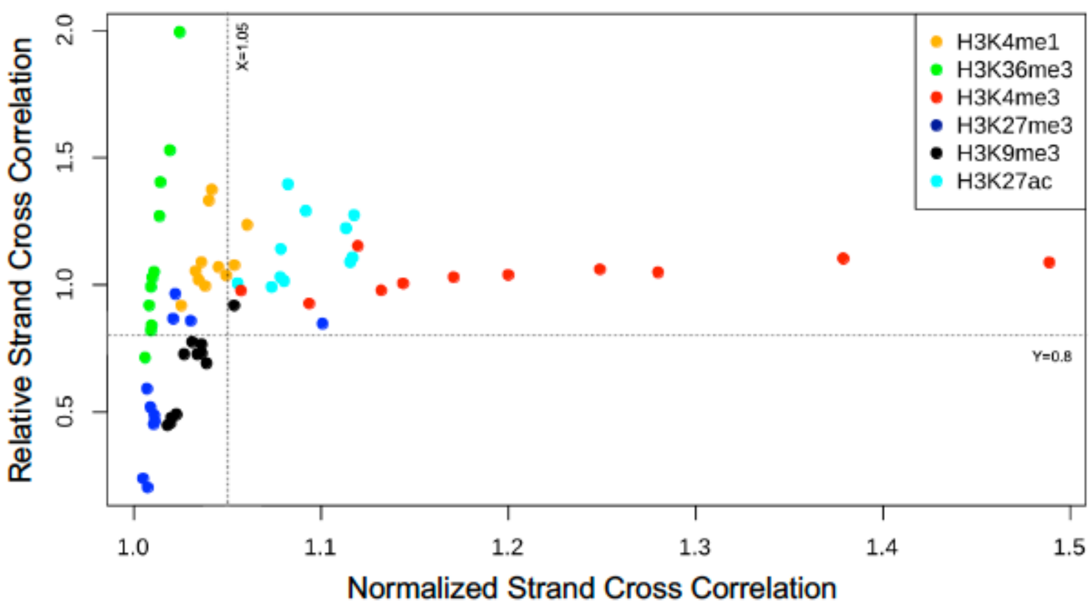

Supplementary Figure 2

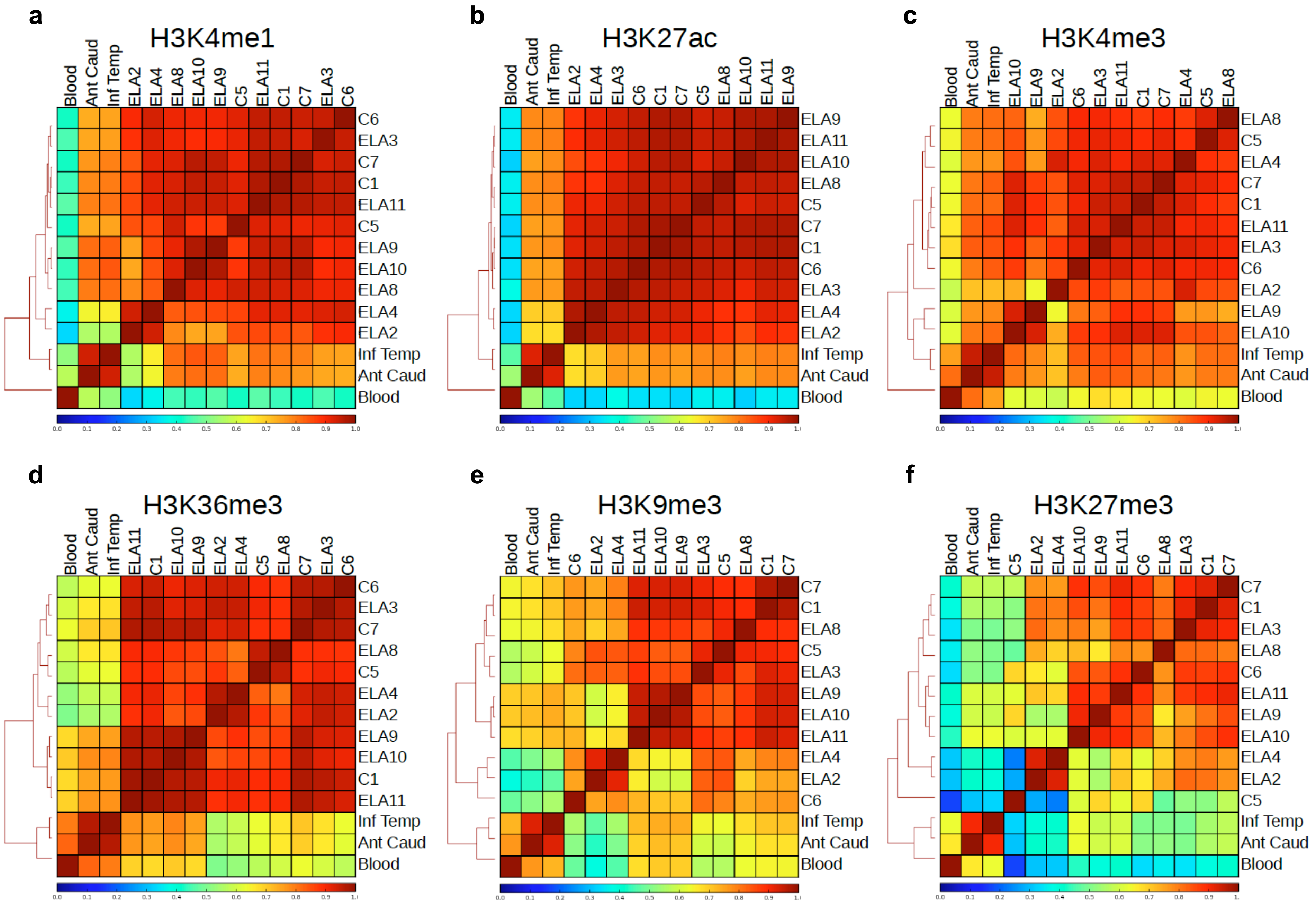

Supplementary Figure 3

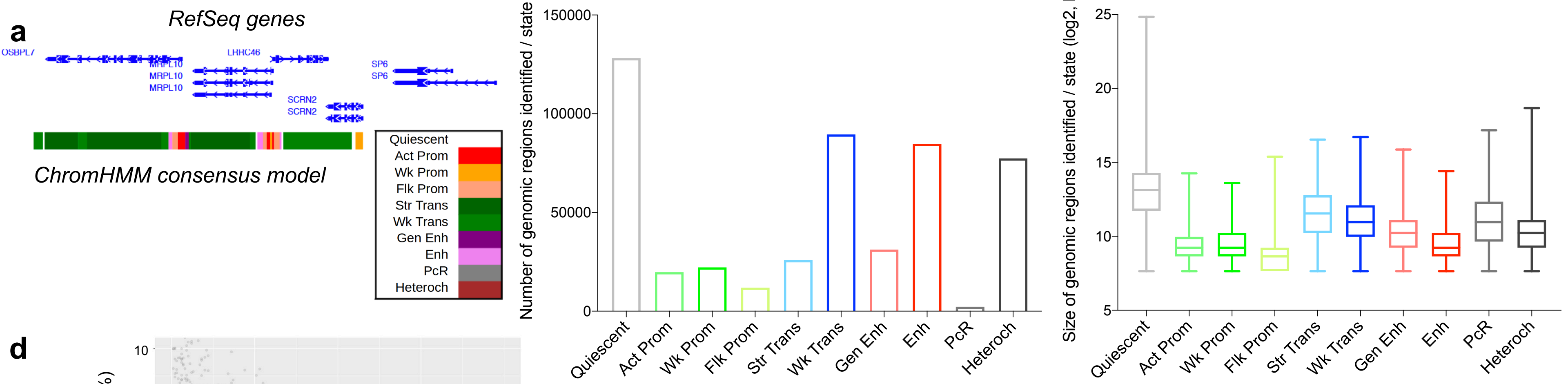

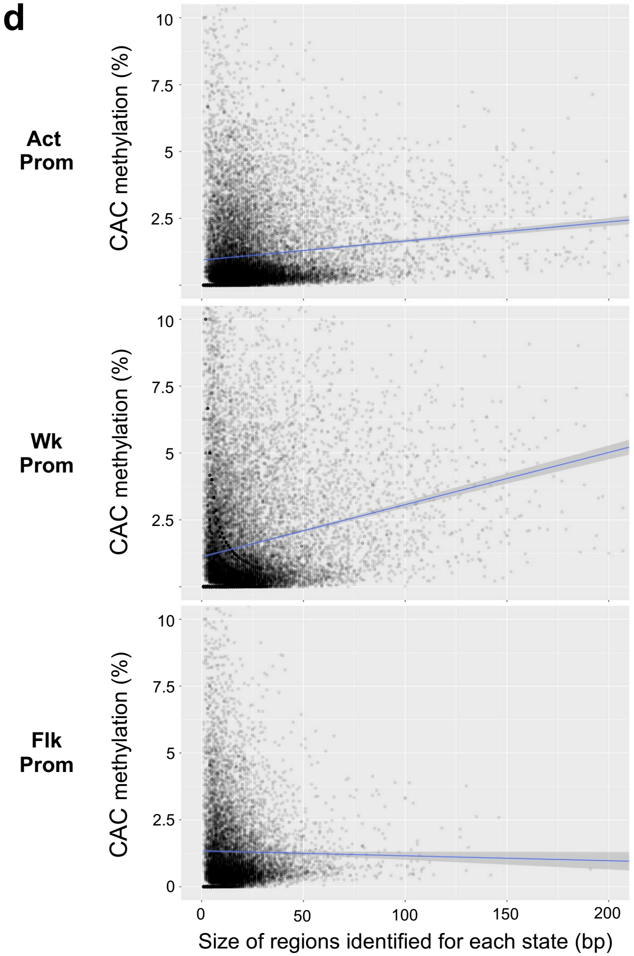

e

Brain amygdala chromatin states

Brain hippocampus chromatin states

	Act Prom	Wk Prom	Flk Prom	Str Trans	Wk Trans	Gen Enh	Enh	Heteroch	PcR	Quiescent
1_TssA	0.29	0.32	0.02	0.00	0.00	0.00	0.00	0.00	0.00	0.00
2_TssAFlnk	0.18	0.04	0.14	0.00	0.00	0.05	0.02	0.00	0.00	0.00
3_TxFlnk	0.01	0.00	0.03	0.00	0.00	0.00	0.00	0.00	0.00	0.00
4_Tx	0.00	0.00	0.00	0.44	0.04	0.01	0.00	0.00	0.00	0.00
5_TxWk	0.00	0.00	0.00	0.15	0.37	0.03	0.05	0.01	0.00	0.02
6_EnhG	0.00	0.00	0.02	0.01	0.00	0.07	0.01	0.00	0.00	0.00
7_Enh	0.01	0.00	0.01	0.00	0.02	0.26	0.26	0.00	0.00	0.00
8_ZNF/Rpts	0.00	0.00	0.00	0.00	0.00	0.00	0.00	0.03	0.02	0.00
9_Het	0.00	0.00	0.00	0.00	0.00	0.00	0.00	0.26	0.02	0.01
10_TssBiv	0.01	0.06	0.00	0.00	0.00	0.00	0.00	0.00	0.02	0.00
11_BivFlnk	0.00	0.03	0.01	0.00	0.00	0.00	0.00	0.00	0.02	0.00
12_EnhBiv	0.00	0.00	0.00	0.00	0.00	0.00	0.00	0.00	0.03	0.00
13_ReprPC	0.00	0.01	0.00	0.00	0.00	0.00	0.00	0.00	0.11	0.01
14_ReprPCWk	0.00	0.00	0.00	0.00	0.00	0.00	0.00	0.01	0.01	0.10
15_Quies	0.00	0.00	0.00	0.00	0.04	0.00	0.01	0.04	0.00	0.77

f

Gastric tissue chromatin states

	Act Prom	Wk Prom	Flk Prom	Str Trans	Wk Trans	Gen Enh	Enh	Heteroch	PcR	Quiescent
1_TssA	0.21	0.29	0.03	0.00	0.00	0.00	0.00	0.00	0.00	0.00
2_TssAFlnk	0.02	0.04	0.05	0.00	0.00	0.00	0.00	0.00	0.01	0.00
3_TxFlnk	0.00	0.00	0.01	0.00	0.00	0.00	0.00	0.00	0.00	0.00
4_Tx	0.00	0.00	0.00	0.24	0.04	0.02	0.01	0.00	0.00	0.00
5_TxWk	0.01	0.00	0.01	0.17	0.25	0.05	0.06	0.01	0.00	0.06
6_EnhG	0.00	0.00	0.01	0.01	0.01	0.02	0.01	0.00	0.00	0.00
7_Enh	0.02	0.02	0.02	0.00	0.02	0.04	0.07	0.00	0.01	0.01
8_ZNF/Rpts	0.00	0.00	0.00	0.00	0.00	0.00	0.00	0.02	0.03	0.00
9_Het	0.00	0.00	0.00	0.00	0.00	0.00	0.00	0.09	0.04	0.01
10_TssBiv	0.00	0.00	0.00	0.00	0.00	0.00	0.00	0.00	0.00	0.00
11_BivFlnk	0.00	0.00	0.00	0.00	0.00	0.00	0.00	0.00	0.01	0.00
12_EnhBiv	0.00	0.00	0.00	0.00	0.00	0.00	0.00	0.00	0.00	0.00
13_ReprPC	0.00	0.00	0.00	0.00	0.00	0.00	0.00	0.00	0.00	0.00
14_ReprPCWk	0.00	0.01	0.00	0.00	0.00	0.00	0.00	0.00	0.04	0.01
15_Quies	0.00	0.00	0.00	0.00	0.05	0.01	0.02	0.05	0.00	0.77

Supplementary Figure 4

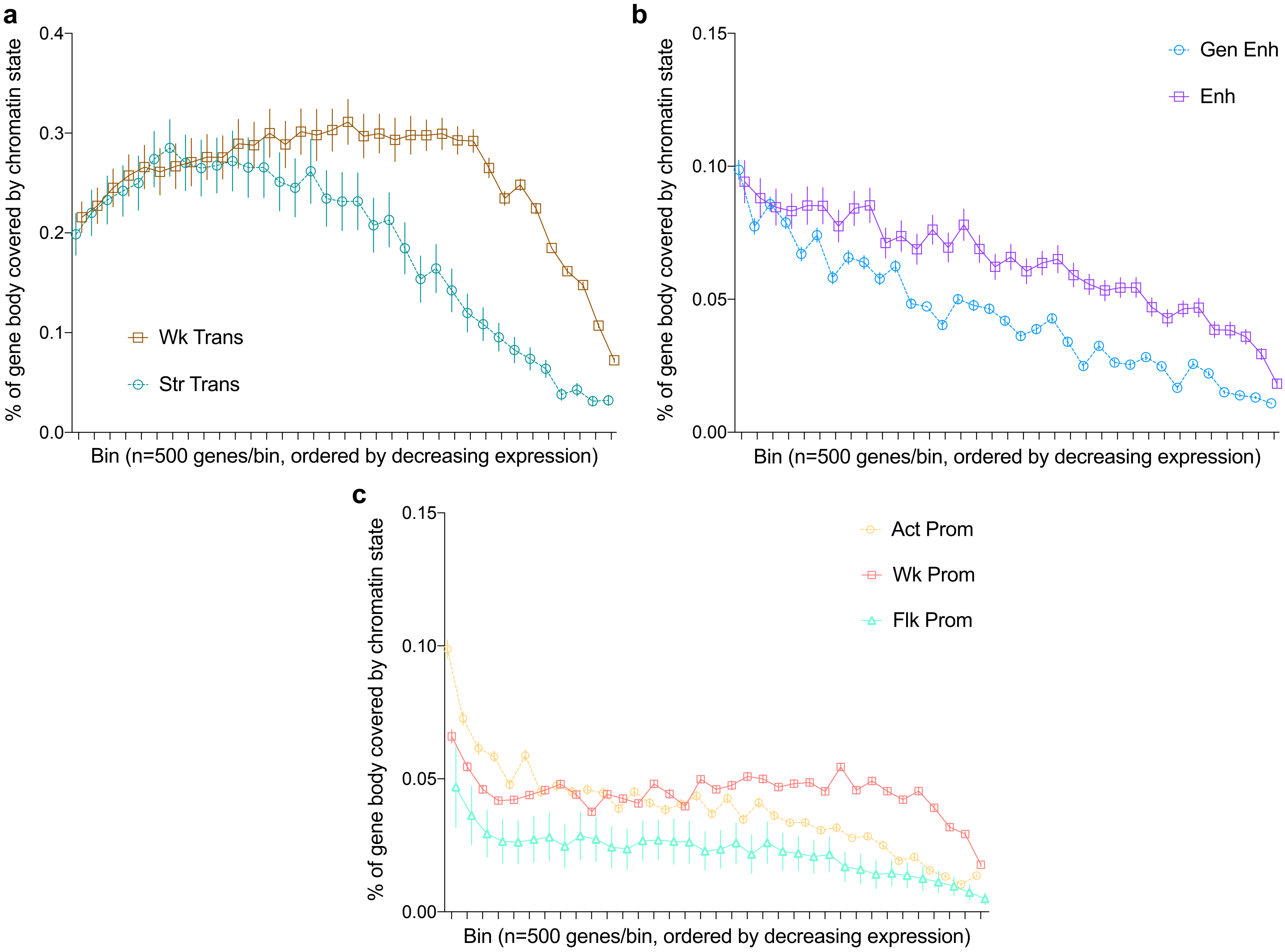

Supplementary Figure 5

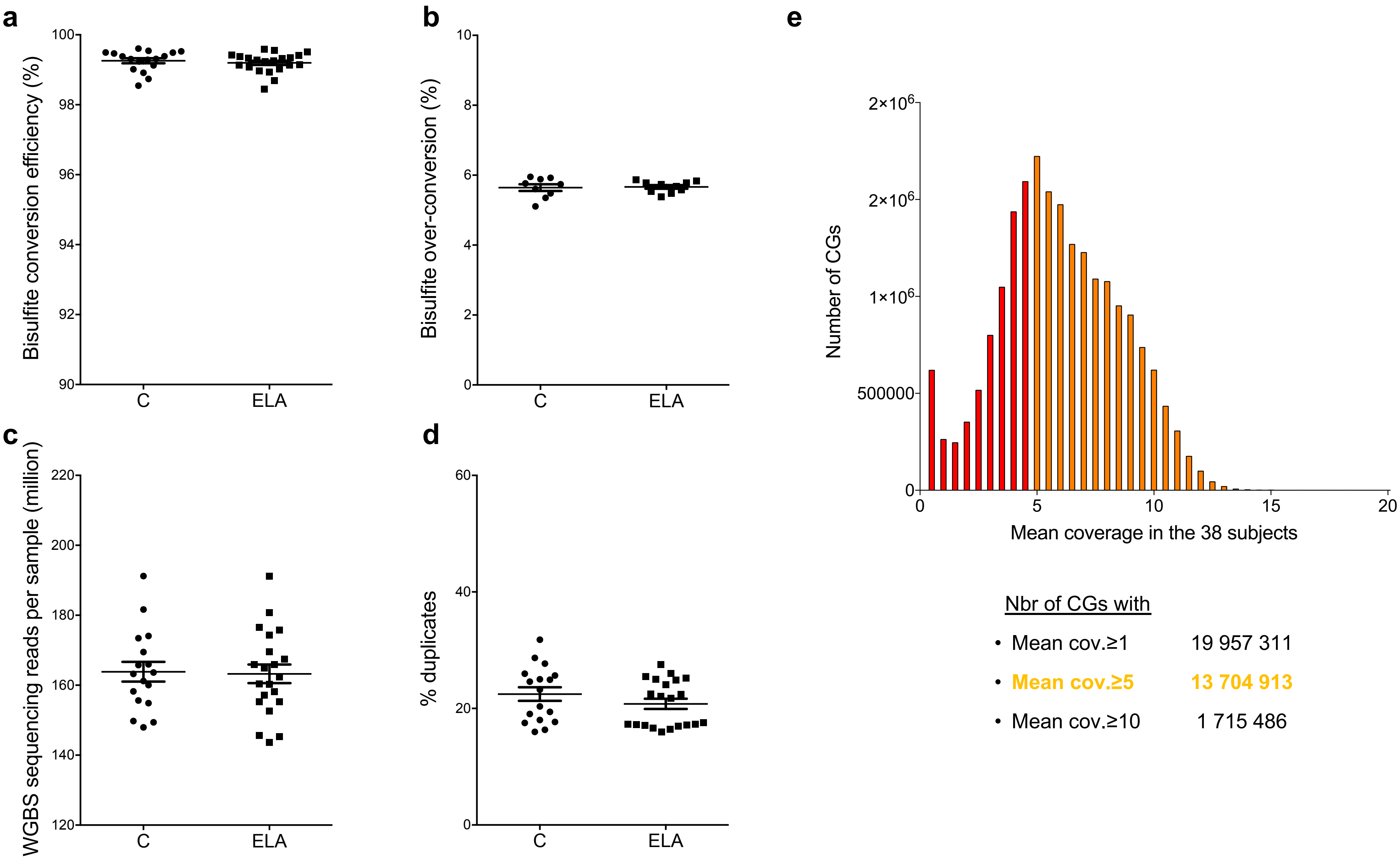

Supplementary Figure 6

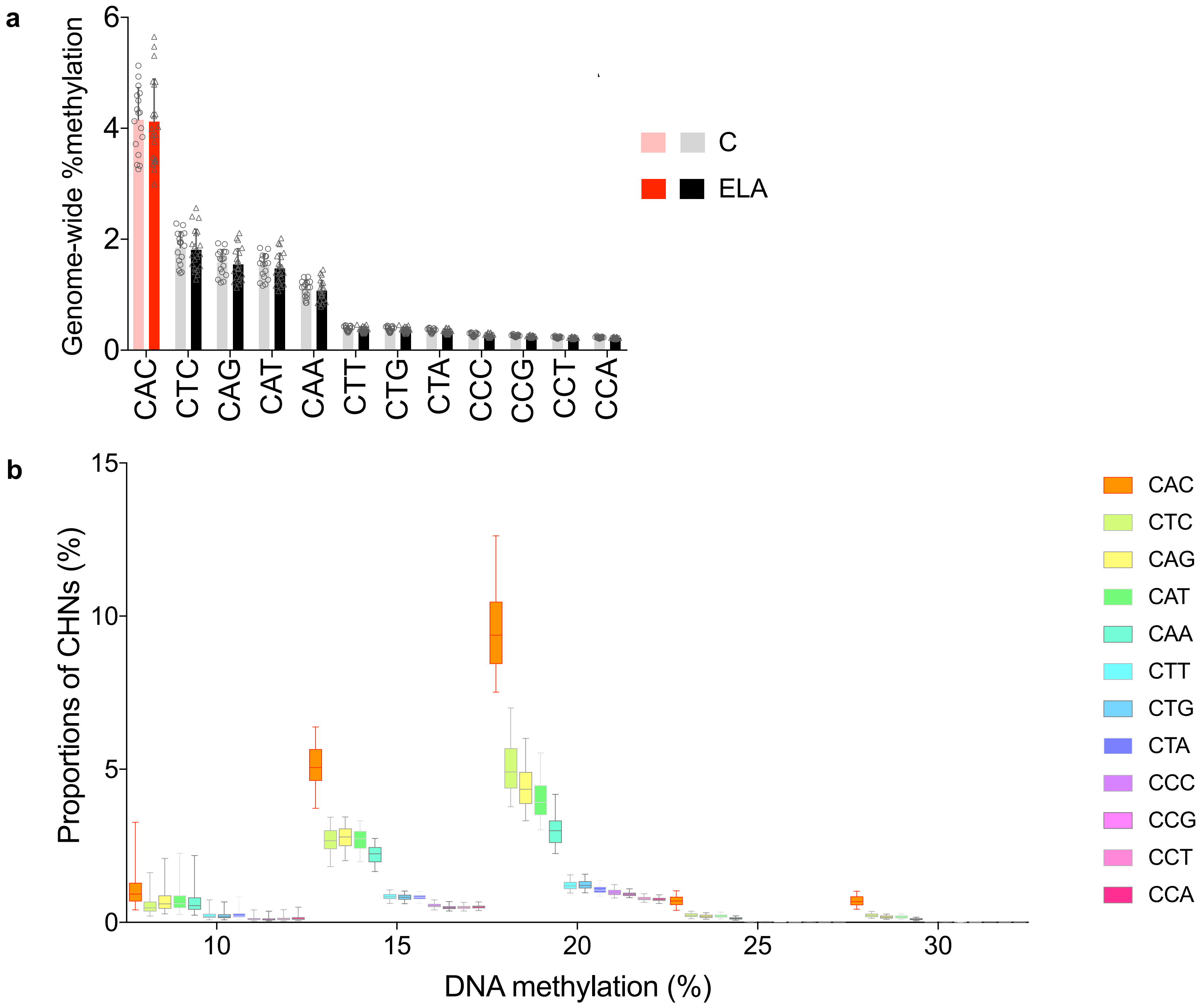

Supplementary Figure 7

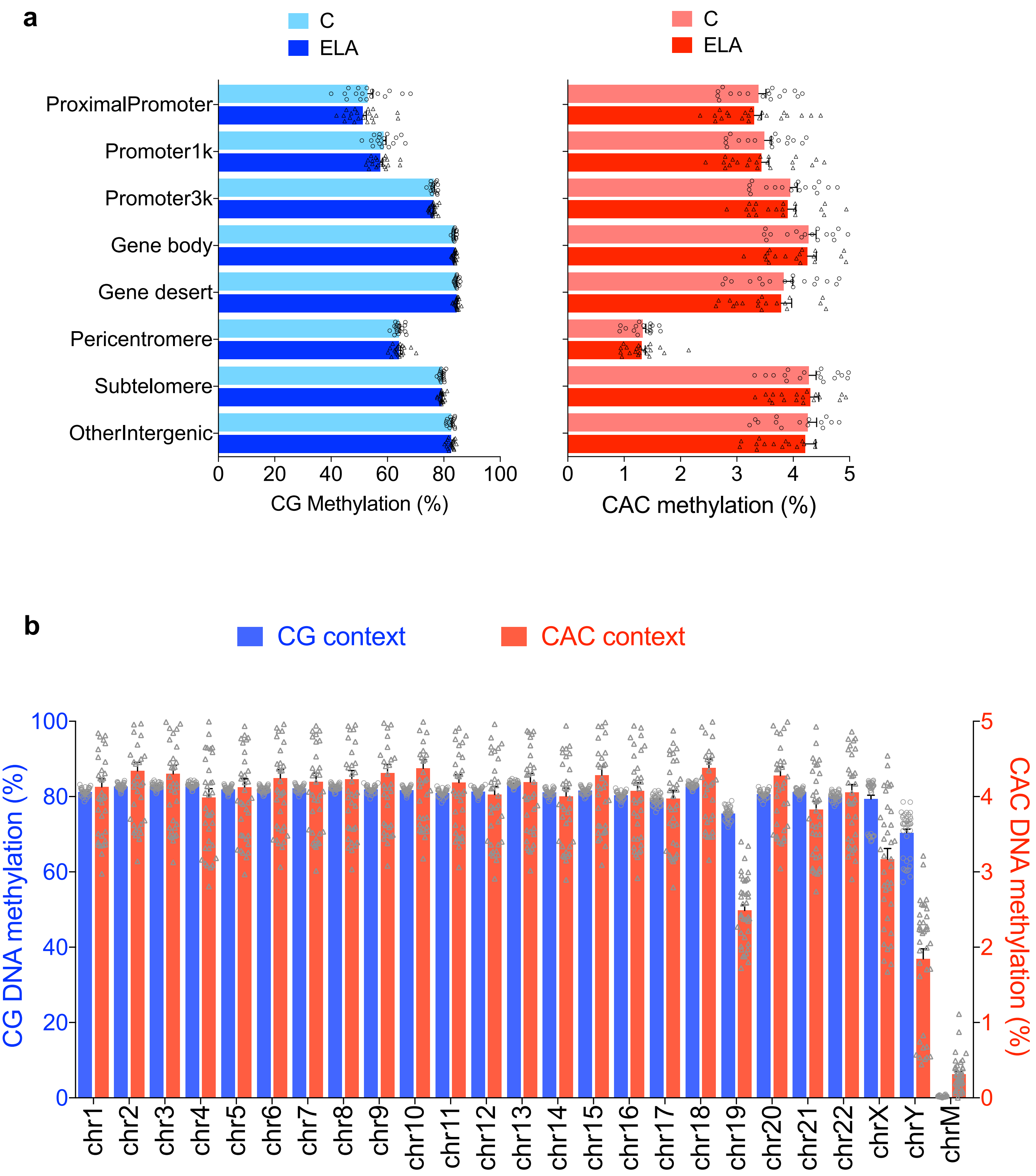

Supplementary Figure 8

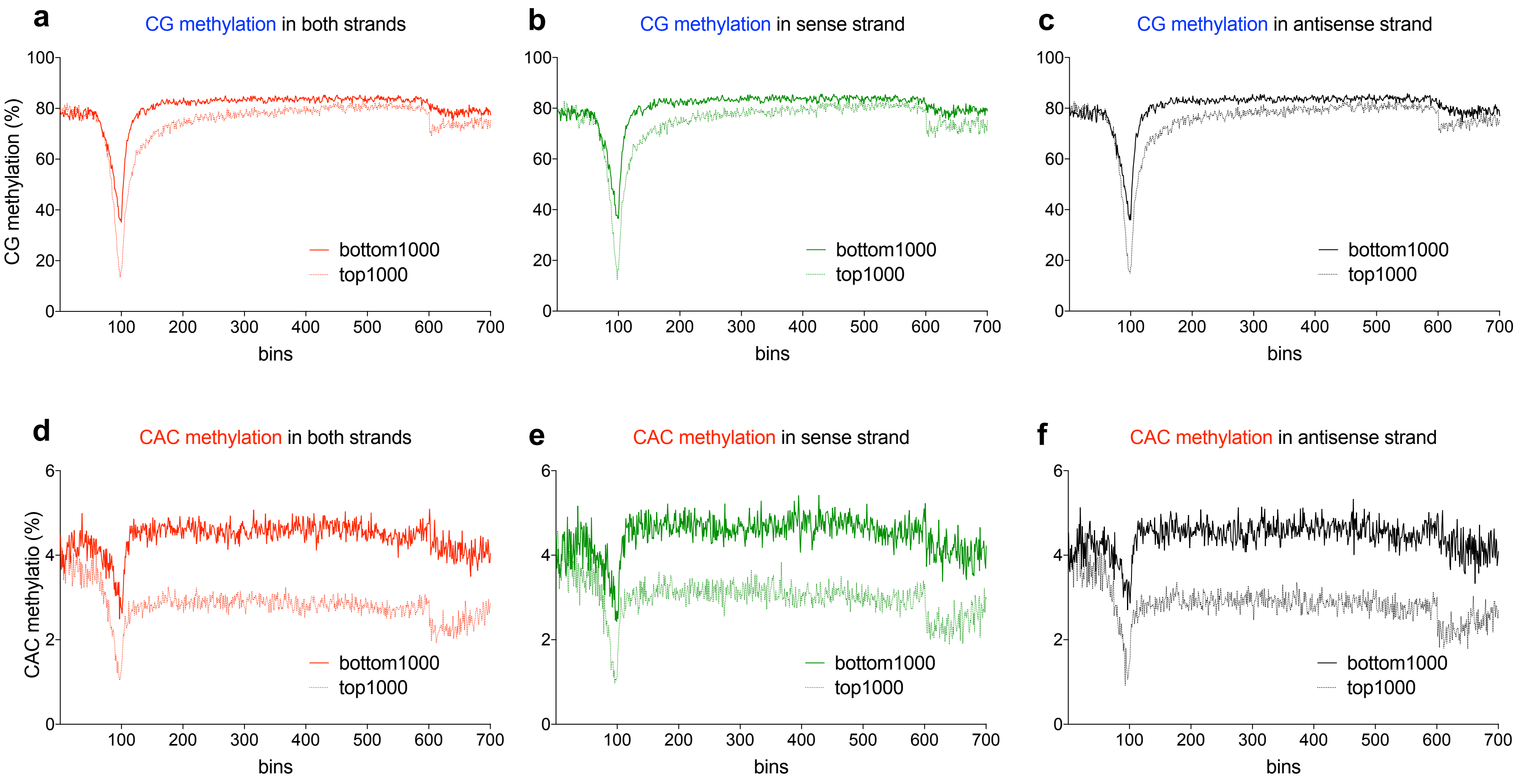

Supplementary Figure 9

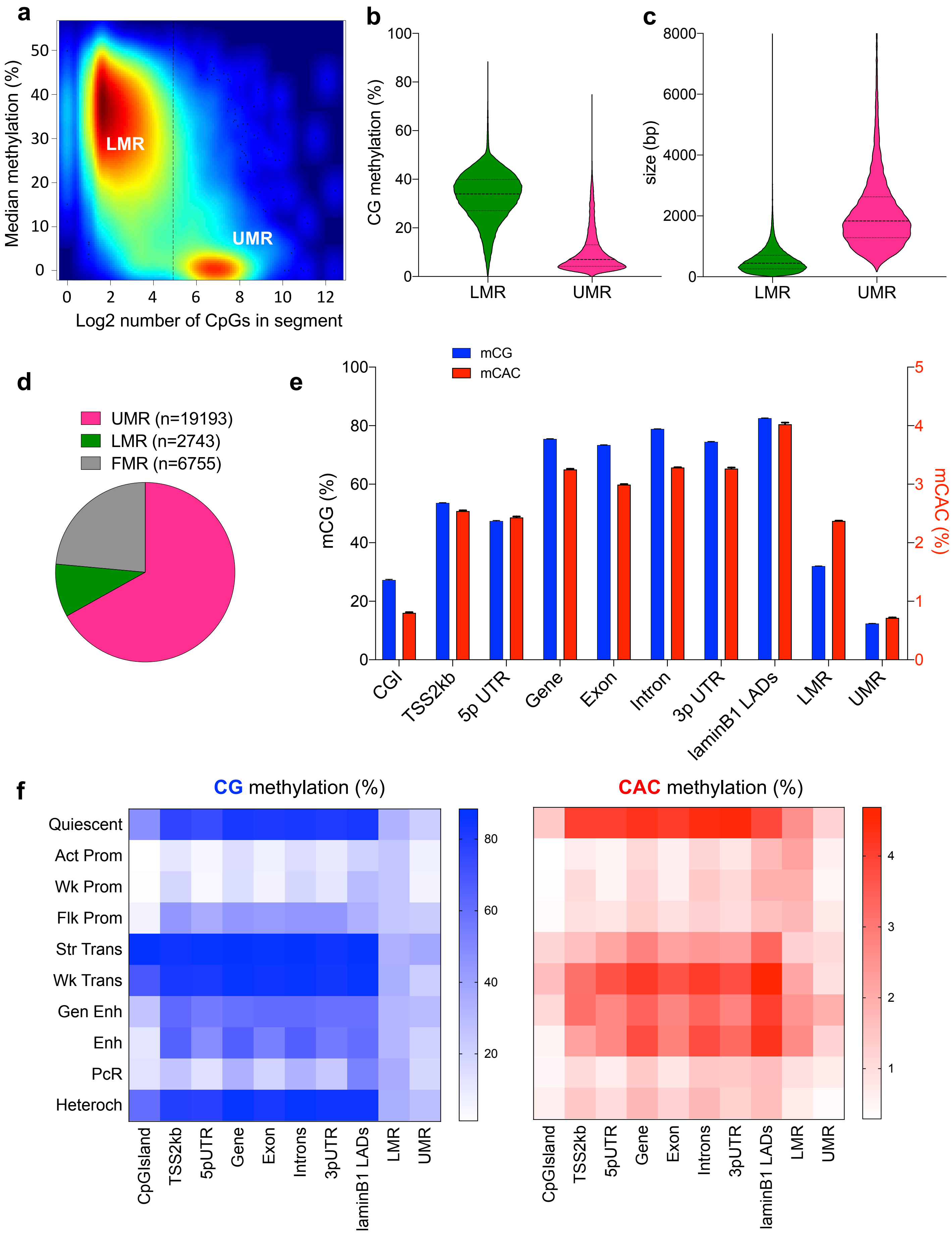

Supplementary Figure 10

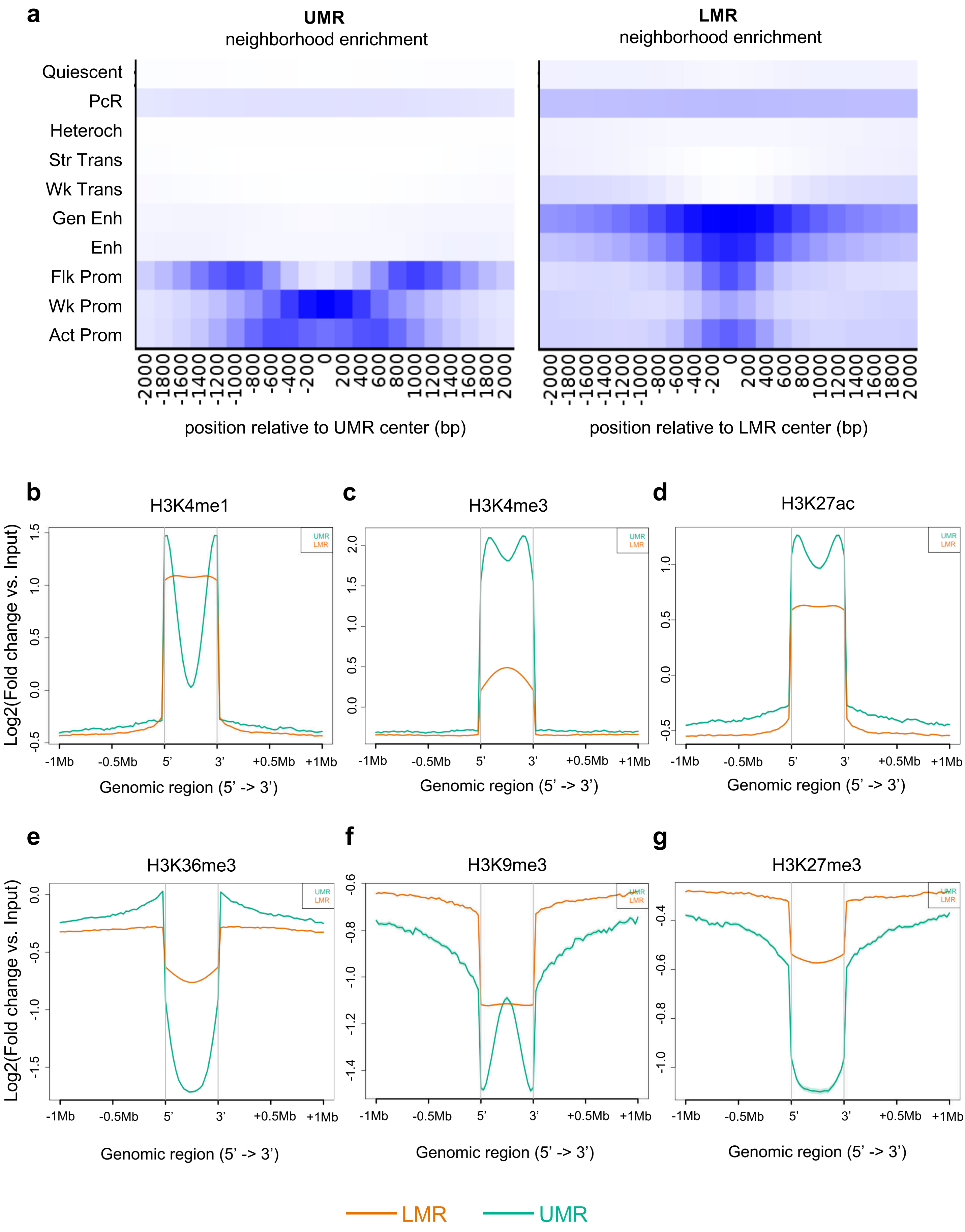

Supplementary Figure 11

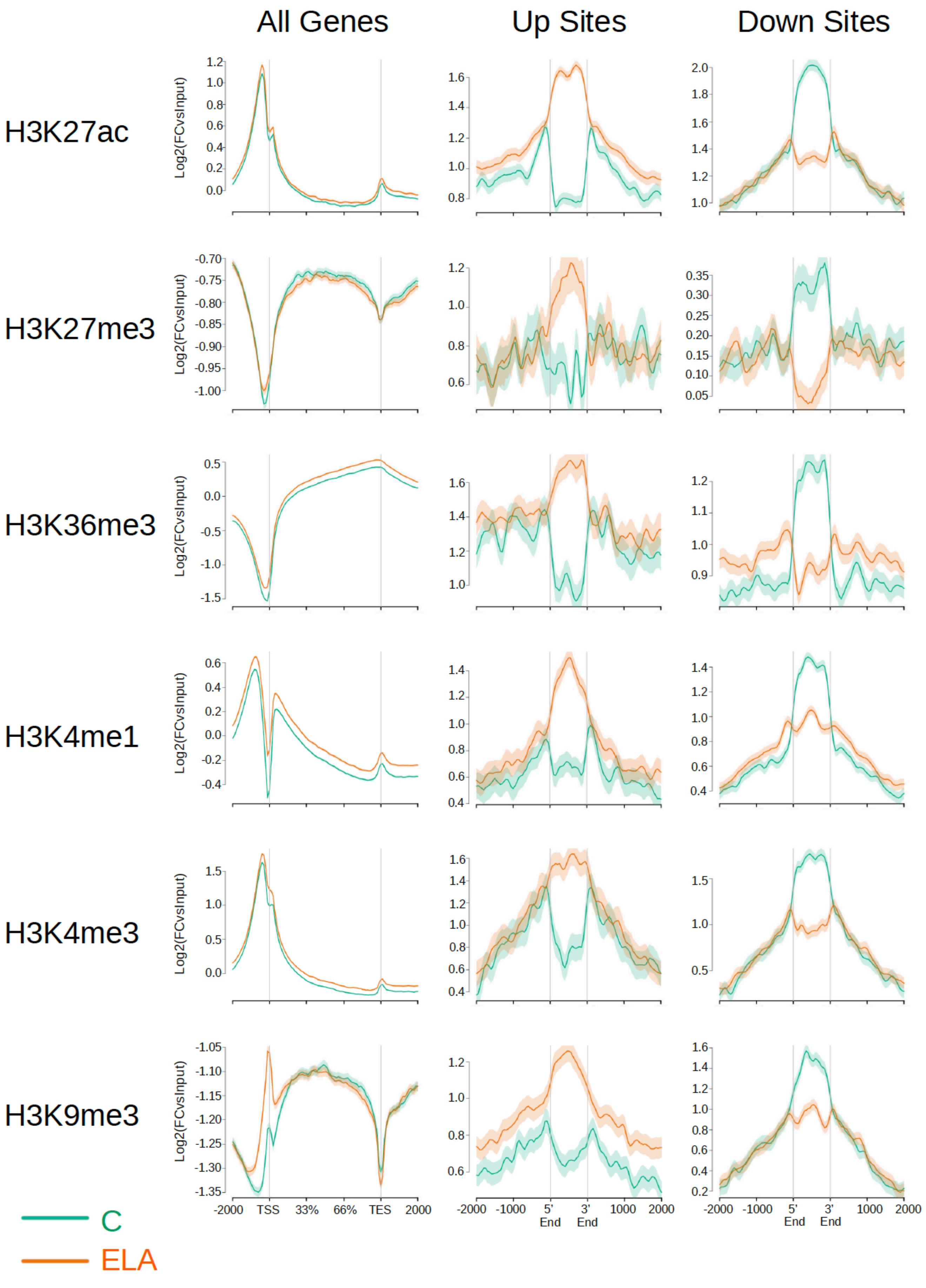

Supplementary Figure 12

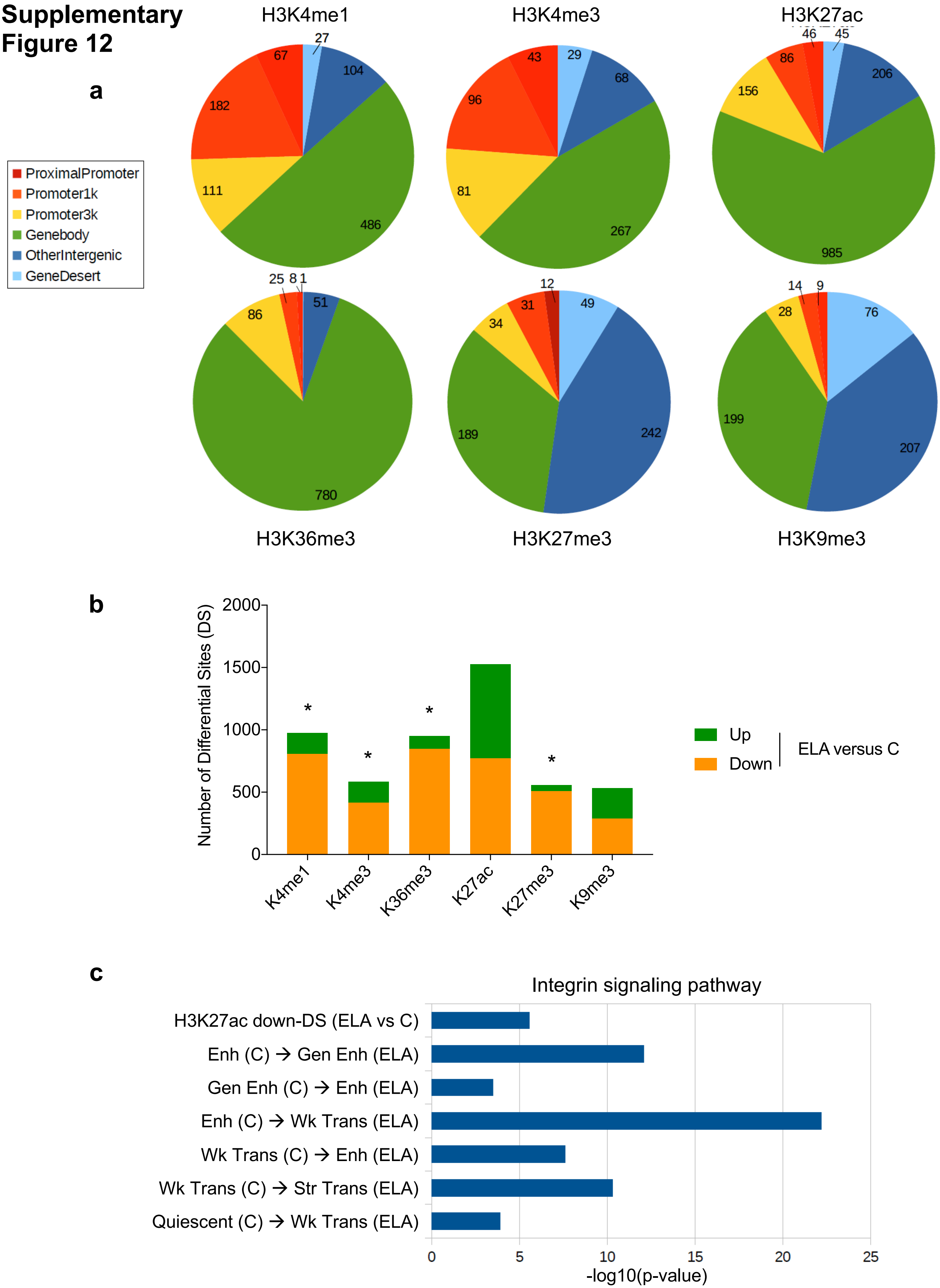

Supplementary Figure 13

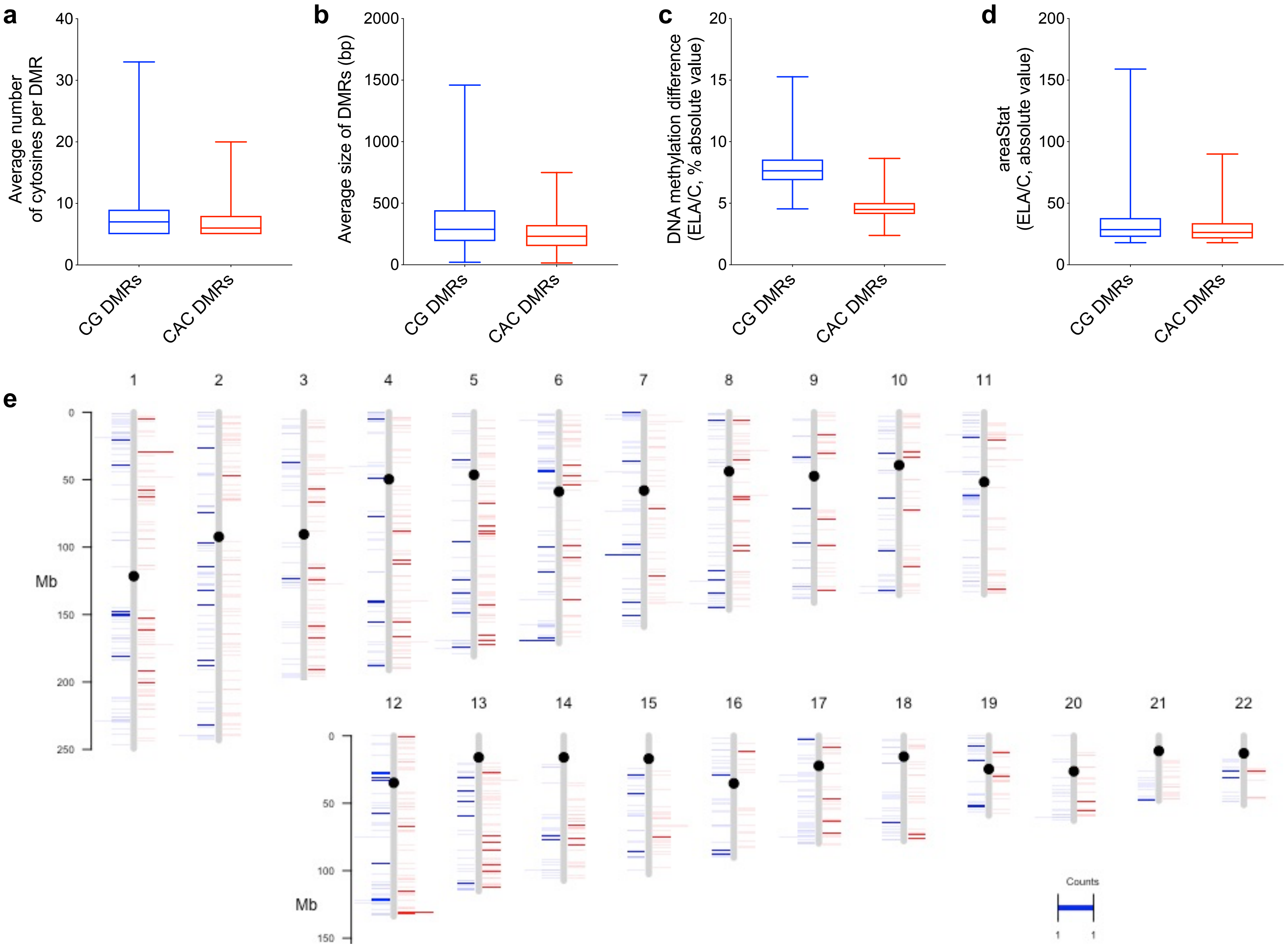

Supplementary Figure 14

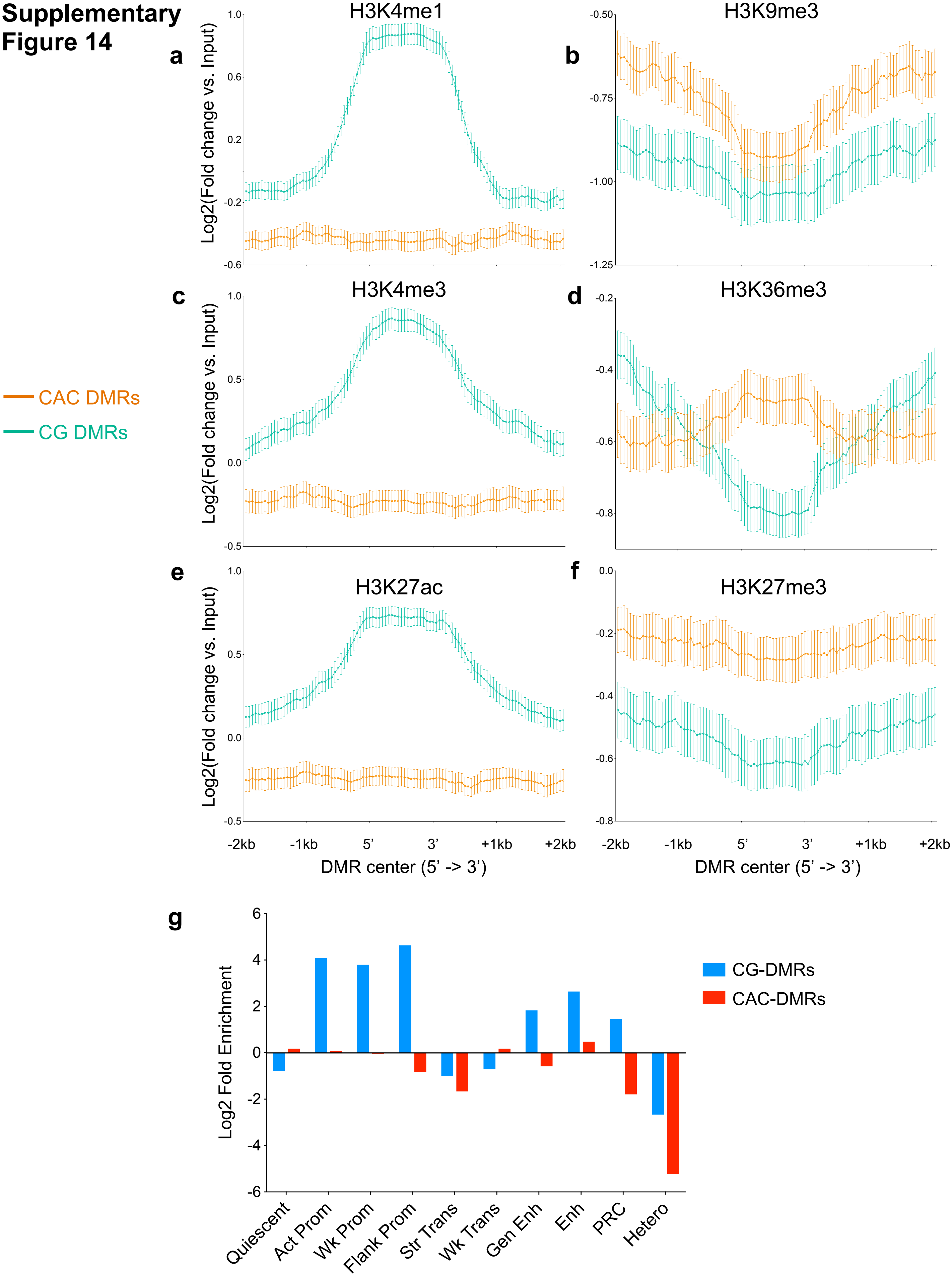

Supplementary Figure 15

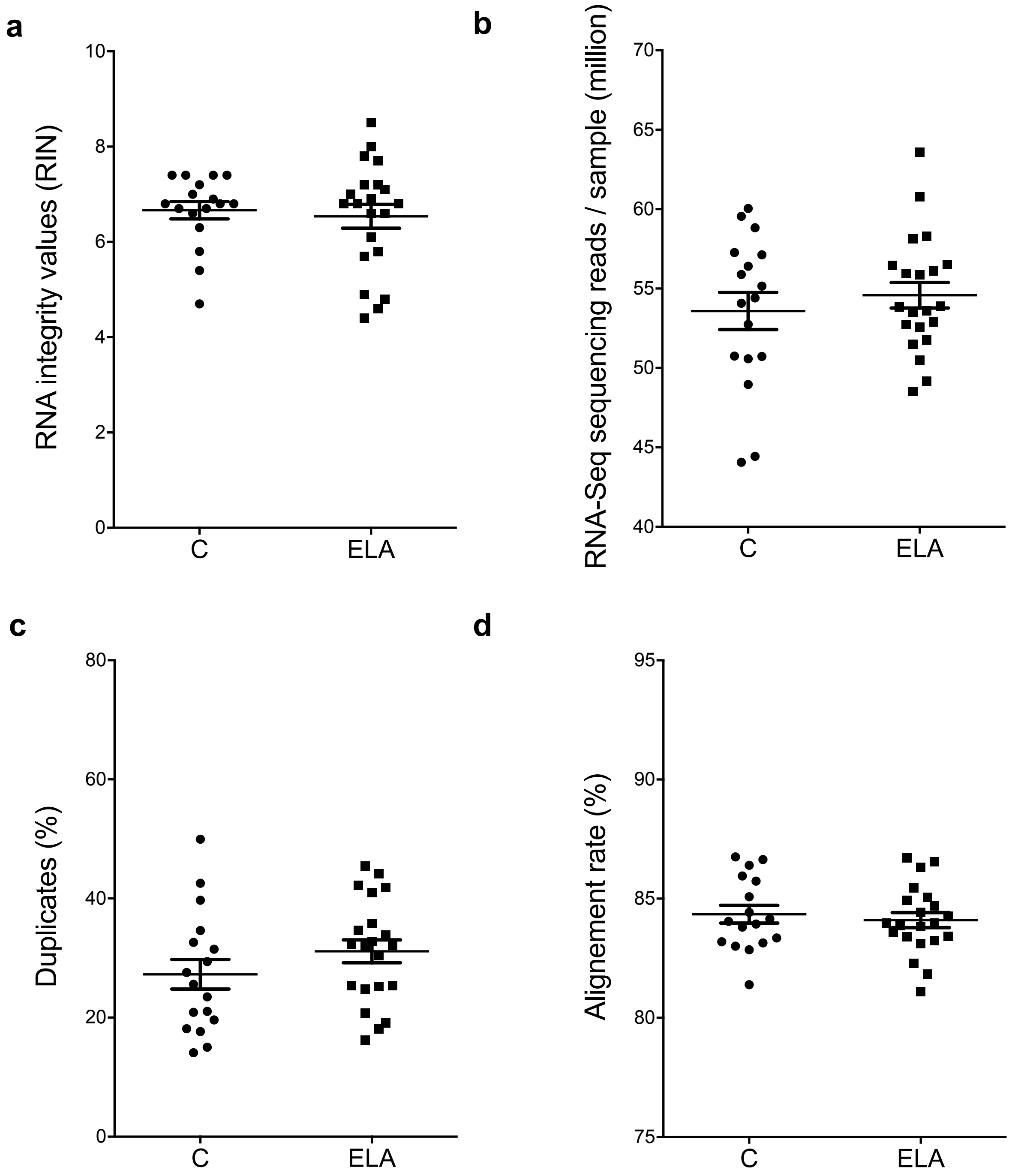

Supplementary Figure 16

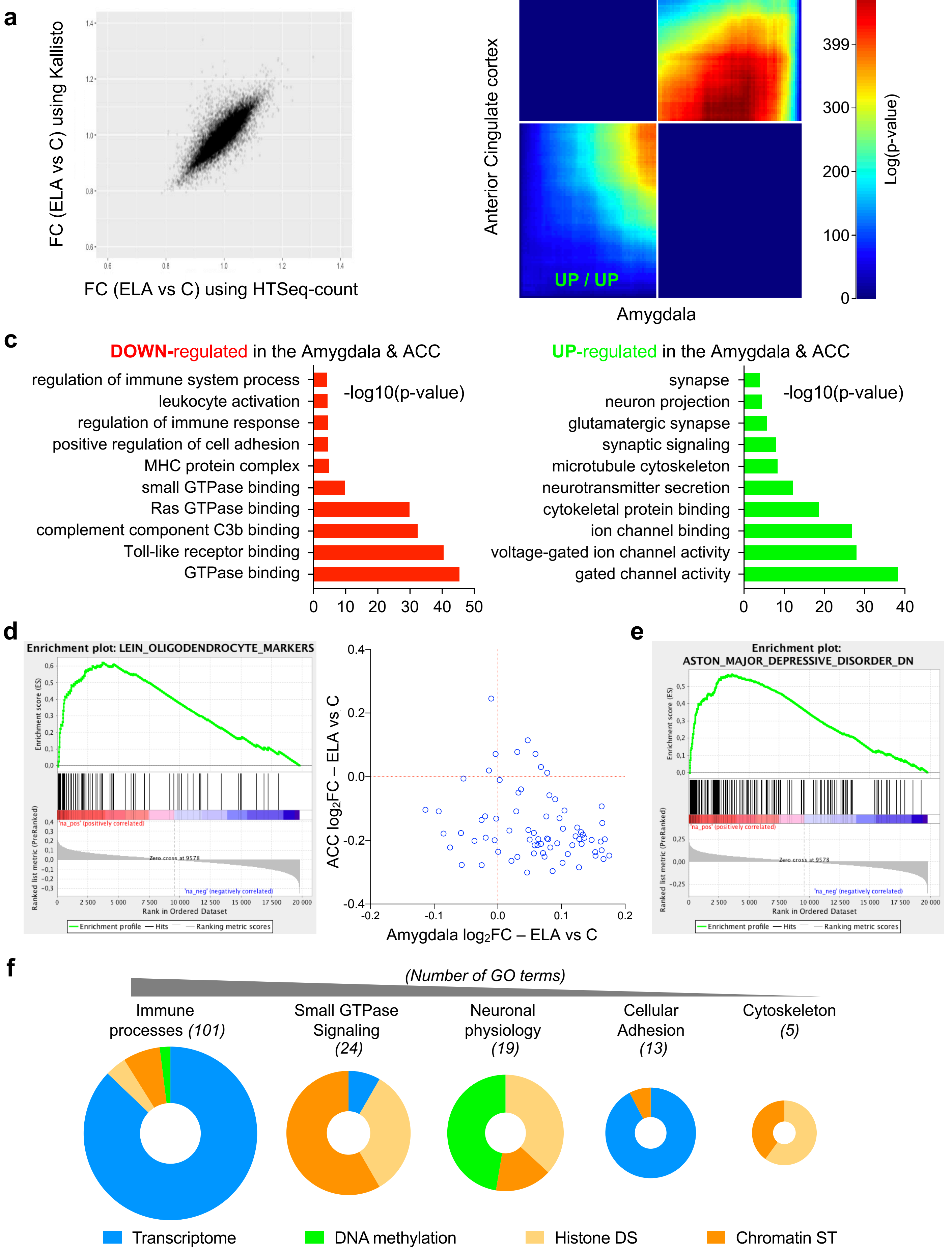

Supplementary Figure 17

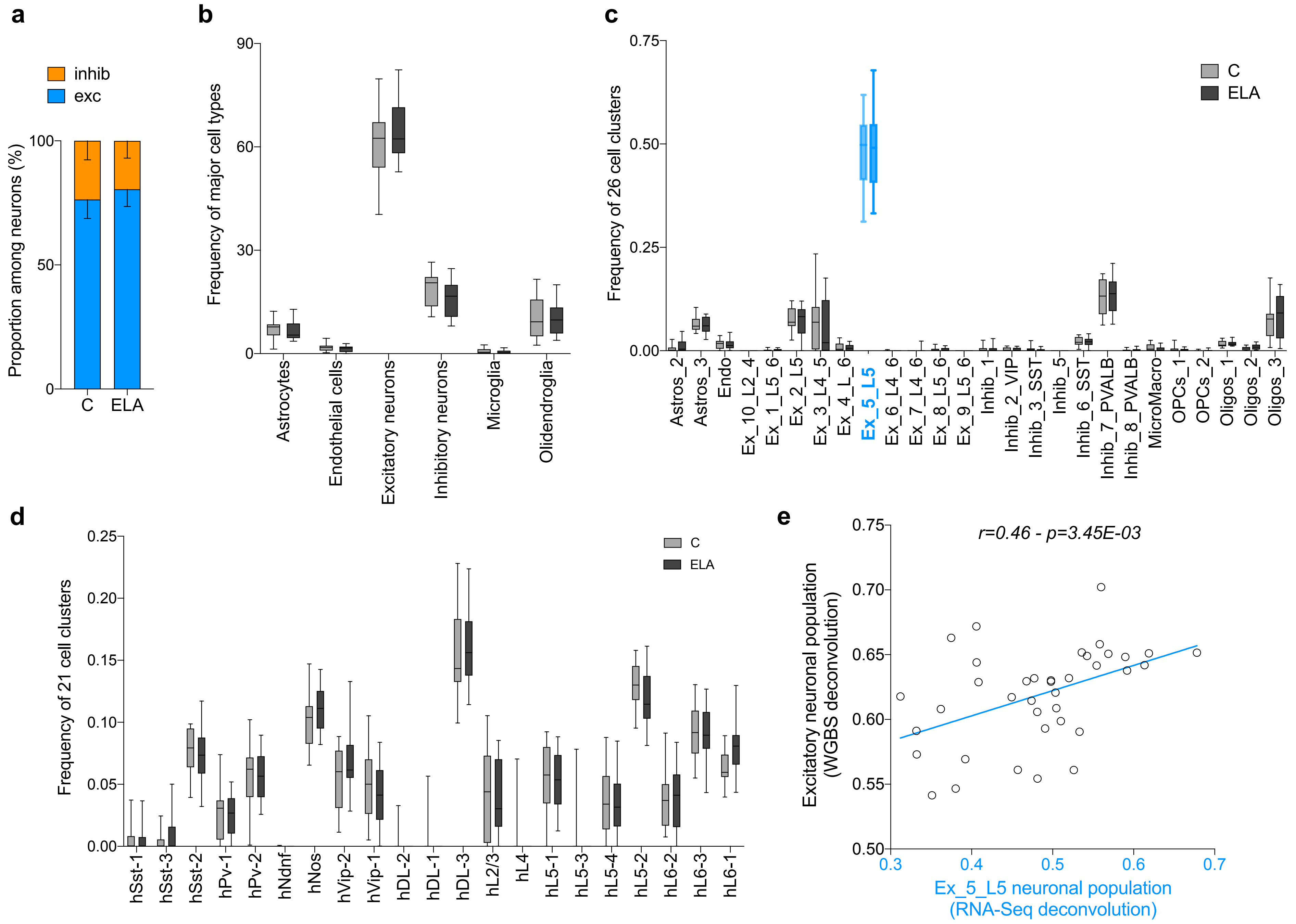

1. Supplementary Tables

Supplementary Table 1. Cohort demographics. Statistical differences were evaluated using Student t-tests.

	Controls (C)	Early-life adversity (ELA)	
N	17	21	p-value
Age (years)	50.3±5.1	42.0±2.6	0.14
Gender M/F	13/4	15/6	0.73
PMI (hours)	25.6±5.2	26.6±4.6	0.91
pH	6.5±0.1	6.6±0.1	0.34

Supplementary Table 2. Type of early-life adversity (ELA) experienced by subjects from the control (C) and ELA groups, and mean of death in the ELA group.

	Neglect (%)	Physical abuse (%)	Sexual abuse (%)	Psychological abuse (%)	*>1 type of abuse (%)*
**C**	0	0	0	0	*0*
**ELA**	42,9	66,7	23,8	19,0	*42,9*

Supplementary Table 3. Tissue pools used in ChIP-Seq experiments.

Group	Pool nbr	Number of subjects pooled	Gender	Tissue available for ChIP (mg)
C	1	6	Male	637
C	5	4	Female	589
C	6	3	Male	383
C	7	4	Male	448
ELA	2	2	Male	397
ELA	3	3	Male	475
ELA	4	2	Male	470
ELA	8	4	Female	449
ELA	9	2	Male	403
ELA	10	3	Male	416
ELA	11	4	Male	525

Supplementary Table 4. Distribution of CG and CAC DMRs among genomic features (expressed in counts or % of total in each context), as assessed using region_analysis.

Count	CG	CAC
Proximal Promoter	28	7
Promoter 1k	132	17
Promoter 3k	138	59
Gene body	287	450
Other intergenic	198	233
Gene desert	12	71
subtelomere	0	2
pericentromere	0	1
TOTAL	795	840

%	CG	CAC
Proximal Promoter	3,52	0,83
Promoter 1k	16,60	2,02
Promoter 3k	17,36	7,02
Gene body	36,10	53,57
Other intergenic	24,91	27,74
Gene desert	1,51	8,45
subtelomere	0	0,24
pericentromere	0	0,12
TOTAL	100	100

Supplementary Table 5. Distribution of CG and CAC DMRs among chromatin states (expressed in counts or % of total in each context), as assessed using ChromHMM data.

Count	CG	CAC
Void	281	644
Act Prom	68	5
Wk Prom	60	5
Flk Prom	37	1
Str Trans	16	12
Wk Trans	43	94
Genic Enh	45	10
Enh	113	30
PRC	8	1
Hetero	5	1
Total	676	803

%	CG	CAC
Void	41,57	80,20
Act Prom	10,06	0,62
Wk Prom	8,88	0,62
Flk Prom	5,47	0,12
Str Trans	2,37	1,49
Wk Trans	6,36	11,71
Genic Enh	6,66	1,25
Enh	16,72	3,74
PRC	1,18	0,12
Hetero	0,74	0,12
Total	100	100

Supplementary Note 1: Analysis codes

4.1 ChIP-Sequencing analyses
#run FASTQC on raw sequencing files:
fastqc FILE

#Use Trimmomatic for adapters and quality
java -jar trimmomatic-0.35.jar SE -phred33 FILE FILE.trim
ILLUMINACLIP:FILENAME:2:30:10 LEADING:3 TRAILING:3 SLIDINGWINDOW:4:25
MINLEN:36

#Align fastQ files against the hg19 genome
bwa aln GENOME_GRCh37 FILE
bwa samse GENOME_GRCh37 FILE
samtools view -Sb FILE

#Use Picard to remove duplicates
java -jar picard.jar MarkDuplicates I=FILE O=FILE.dedup M=dedupmetrics.txt
VALIDATION_STRINGENCY=LENIENT REMOVE_DUPLICATES=true

#Use DeepTools computeGCBias and correctGCbias functions to correct the GC bias
computeGCBias -b FILE --effectiveGenomeSize 2451960000 -g hg19.2bit -l 200 -p 3 -freq
Gcbias.txt
correctGCBias -b FILE --effectiveGenomeSize 2451960000 -g hg19.2bit -o FILE.GCcor.bam
-p 3 -freq Gcbias.txt

#Use PhantomPeakQualTools to compute the Normalized and Relative Strand Cross
Correlations (FigS1b)
Rscript run_spp.R -c=FILE -p=10 -odir=DIRECTORY -savp -
out=PhantomPeak_measureSummary.txt

#Use DeepTools bamCompare to create bigwig files (Fig1a)
bamCompare --ratio subtract -p 10 --normalizeUsingRPKM -e 100 -b1 SIGNAL_FILE -b2
INPUT_FILE -of bigwig -o FILE.bw

#Use ngs.plot to visualize histone profiles around genes (Fig1c)
ngs.plot.r -G hg19 -R genebody -L 2000 -FL 200 -C CONFIG_FILE -O DIRECTORY

#Do hierarchical clustering based on the signals of our files (Fig1b)
multiBigwigSummary bins -b FILE_1.bw FILE_2.bw ... -out SUMMARY.npz
plotCorrelation -in SUMMARY.npz -c pearson -p heatmap -o PLOT

#Do hierarchical clustering based on the signals of our files and histone profiles from the
anterior caudate, blood cells and brain inferior temporal tissue (FigS2)
multiBigwigSummary bins -b ANT_CAUD.bw BLOOD.bw INF_TEMP.bw FILE_1.bw
FILE_2.bw ... -out SUMMARY.npz
plotCorrelation -in SUMMARY.npz -c pearson -p heatmap -o PLOT

#Calculate number of histone reads at genebodies (H3K36me3, H3K27me3, H3K9me3,
H3K4me1) or TSS (H3K4me3, H3K27ac), organized by gene expression levels. These are
then normalized against input levels before plotting (see below)
bedtools multicov -bed GENEBODY.bed -bams H3K36me3.bam H3K27me3.bam
H3K9me3.bam H3K4me1.bam INPUT.bam > GENEBODY_MARKS.csv
bedtools multicov -bed TSS.bed -bams H3K4me3.bam H3K27ac.bam INPUT.bam >
TSS_MARKS.csv

#Plot rolling averages of histone read number at genes ranked by gene expression (data points generated using the "zoo" R package) (Fig1d)

```
	library(zoo)
	avg.MARK<-rollmean(zoo(data$MARK),1000)
```

#Use DiffReps to identify differential sites, done for each histone mark independently

```
diffReps.pl -gn hg19 --frag 200 -me nb -tr SIGNAL_ELA1 SIGNAL_ELA2 ... -co
SIGNAL_CONTROL1 SIGNAL_CONTROL2 ... --btr INPUT_ELA1 INPUT_ELA2 ... --bco
INPUT_CONTROL1 INPUT_CONTROL2 ... -re FILE.txt
```

#Annotate each DS with region_analysis (FigS12a)

```
region_analysis.pl -r -d ensembl -g hg19 -i FILE
```

#Create chromatin state maps with ChromHMM (Fig1e)

```
java -jar $CHROMHMM BinarizeBed -peaks hg19.txt DIRECTORY cellmarkfile.txt
DIRECTORY #with cellmarkfile organized as per chromHMM standard instructions, with cell
= ELA or Control, mark = 6 histone marks
java -jar $CHROMHMM LearnModel DIRECTORY DIRECTORY 10 hg19
bedtools multiinter -i CONTROL1_STATE1.bed CONTROL2_STATE1.bed
ELA1_STATE1.bed ELA2_STATE1.bed ... | awk '($4>="6") {print $0}' | bedtools merge -i |
cut -f1,2,3 > CHARACTERIZATION_STATE1.bed
```

#Create a consensus chromatin state map for each group, and making 200bp windows (FigS3q)

```
bedtools multiinter -i CONTROL1_STATE1.bed CONTROL2_STATE1.bed | awk '($4>="3")
{print $0}' | bedtools merge -i | cut -f1,2,3 > CONTROL_STATE1.bed
bedtools multiinter -i ELA1_STATE1.bed ELA2_STATE1.bed | awk '($4>="5") {print $0}' |
bedtools merge -i | cut -f1,2,3 > ELA_STATE1.bed
bedtools makewindows -b CONTROL_STATE1.bed -w 200 >
CONTROL_STATE1_windowed.bed
bedtools makewindows -b ELA_STATE1.bed -w 200 > ELA_STATE1_windowed.bed
```

#Compare each state of C to ELA (perl script) to identify state transitions

```
	#! /usr/bin/perl
	use warnings;
	use strict;

	my $k = 1;
	for (my $i = 1; $i<=10; $i++) {
	   for (my $j = 1; $j<=10; $j++) {
	   print $k." - Generating file CONTROL".$i."_to_ELA".$j.".bed...\n";
	   system("bedtools intersect -a CONTROL_STATE".$i."_windowed.bed -b
ELA_STATE".$j."_windowed.bed > C".$i."_to_ELA".$j.".bed");
	   $k++;
	   }
	}
```

#Annotate each ST with region_analysis (Fig4b)

```
region_analysis.pl -r -d ensembl -g hg19 -i FILE
```

#Compute jaccard coefficient between our chromHMM states and publically available hippocampus and gastric chromatin states (FigS3e-f)

```
bedtools jaccard -a CHARACTERIZATION_FILE -b PUBLIC_FILE
```

```
#Visualize histone profiles at DMRs with ngs.plot (Fig6c-d)
ngs.plot.r -G hg19 -R bed -L 2000 -FL 200 -C CONFIG_FILE -O PLOT

#Calculate the number of DMRs intersecting with each type of chromatin state (Fig6e)
bedtools intersect -b CONSENSUS_STATE1.bed -a DMR.bed -f 0.50 | wc -l

#Visualize the neighborhood enrichment of chromatin states around UMRs and LMRs
(FigS10a)
java -jar ChromHMM.jar NeighborhoodEnrichment -nostrand -m FILE FILE.bed UMR.bed
PLOT

#Visualize histone profiles around UMRs and LMRs with ngsplot (FigS10b-g)
ngs.plot.r -G hg19 -R bed -L 1000000 -FL 200 -C CONFIG_FILE -O PLOT

#Visualize histone profiles around DS with ngsplot (FigS11)
ngs.plot.r -G hg19 -R bed -L 2000 -FL 200 -C CONFIG_FILE -O PLOT

#Call peaks using MACS2. For K36me3, K4me1, K27me3 and K9me3 the broad and
nomodel parameters are used. Peaks across the samples are merged. The number of DS
having an intersection with peaks were then identified
macs2 callpeak -t SIGNAL_FILE.bam -f BAM -g hs -n NAME -c INPUT_FILE.bam
macs2 callpeak -t SIGNAL_FILE.bam -f BAM -g hs -n NAME -c INPUT_FILE.bam --nomodel
--broad
ls *MARK | parallel "cat {} | cut -f1,2,3 | bedtools sort -i - | bedtools merge -i -"
bedtools intersect -u -a DS_FILE -b PEAK_FILE

## Compute statistics for enrichment of MACS peaks vs chromHMM DS using regioneR:
> library(regioneR)
> result_MARK_DS <- overlapPermTest(MARK_DS, MARK_MACS, ntimes=100000,
genome="hg19", count.once=TRUE)
```

4.2 WGBS Analyses

See Johnson et al, Current Protocols in Molecular Biology (2012) 21.23.1-21.23.28 (DOI: 10.1002/0471142727.mb2123s99) for details.

```
# Create C-to-T and G-to-A converted reference genomes
cat hg19.fa | perl -pe ’if(!(/^>/)){ tr/cC/tT/ }’ > hg19.ct.fa
cat hg19.fa | perl -pe ’if(!(/^>/)){ tr/gG/aA/ }’ > hg19.ga.fa

# Index the converted reference genome sequence
bin/bwa-0.5.9/bwa index -a bwtsw data/reference/hg19.ct.fa.gz
bin/bwa-0.5.9/bwa index -a bwtsw data/reference/hg19.ga.fa.gz

# Clip off first base of read 1 (same for read 2):
cat dat/reads/bsseq 1 sequence.fq \
| perl -pe ‘$seq header=$ ;
$seq=<>;
$seq=substr($seq, 1);
$qual header=<>;
$qual=<>;
$qual=substr($qual, 1);
$ ="$seq header$seq$qual header$qual"’ \
> data/reads/bsseq 1 sequence.trim.fq

# C-to-T convert read 1 (similar for G-to-A conversion of read 2):
```

```
cat data/reads/bsseq 1 sequence.trim.fq \
| perl -pe '$seq header=$ ;
$seq=<>;
$qual header=<>;
$qual=<>;
$seq =~ tr/cC/tT/;
$ ="$seq header$seq$qual header$qual"' \
> data/reads/bsseq 1 sequence.ct.fq

# Map reads to the converted reference genome (same for reverse strand):
bwa-0.5.9/bwa aln -I -t 8 -q 20 \
data/reference/hg19.ct.fa.gz \
data/reads/bsseq 1 sequence.ct.fq.gz \
> results/mapping/bsseq 1 sequence.ct.hg19.ct.sai

bin/bwa-0.5.9/bwa aln -I -t 8 -q 20 \
data/reference/hg19.ct.fa.gz \
data/reads/bsseq 2 sequence.ga.fq.gz \
> results/mapping/bsseq 2 sequence.ga.hg19.ct.sai

# Generate SAM file of forward-strand mapping containing the unconverted read
sequence and convert SAM to BAM formatted file (same for reverse strand):
bin/bwa-0.5.9/bwa sampe
data/reference/hg19.ct.fa.gz \
results/mapping/bsseq 1 sequence.ct.hg19.ct.sai \
results/mapping/bsseq 2 sequence.ga.hg19.ct.sai \
data/reads/bsseq 1 sequence.trim.fq.gz \
data/reads/bsseq 2 sequence.trim.fq.gz \
| samtools view -bS - \
> results/mapping/bsseq.hg19.forward.bam

# Clip 3' end of overlapping read pairs in forward and reverse strand mappings:
java -jar bin/nxtgen-utils-0.1/NxtGenUtils.jar \
ClipOverlappingReadPairs \
-i $PWD/results/mapping/bsseq.hg19.forward.bam \
-o $PWD/results/mapping/bsseq.hg19.forward.clipped.bam \
-s $PWD/results/mapping/bsseq.hg19.forward.clipping.stats

java -jar bin/nxtgen-utils-0.1/NxtGenUtils.jar \
ClipOverlappingReadPairs \
-i $PWD/results/mapping/bsseq.hg19.reverse.bam \
-o $PWD/results/mapping/bsseq.hg19.reverse.clipped.bam \
-s $PWD/results/mapping/bsseq.hg19.reverse.clipping.Stats

# Sort read mappings by read name:
bin/samtools-0.1.18/samtools sort -n \
results/mapping/bsseq.hg19.forward.clipped.bam \
results/mapping/bsseq.hg19.forward.clipped.readname.sorted
bin/samtools-0.1.18/samtools sort -n \
results/mapping/bsseq.hg19.reverse.clipped.bam \
results/mapping/bsseq.hg19.reverse.clipped.readname.sorted

# Identification of DMR was conducted as described in the methods section, using BSmooth
at default settings for smoothing:
```

```
# Remove clonal reads, read pairs not mapped at the expected distance based on the
library insert size, and reads with a mapping quality score below 20:
bin/samtools-0.1.18/samtools view -b -q 20 -f 2 -F 1024
results/mapping/bsseq.hg19.forward.clipped.processed.dupmark.bam \
-o results/mapping/bsseq.hg19.forward.clipped.processed.dupmark.filtered.1.bam

bin/samtools-0.1.18/samtools view -b -q 20 -f 2 -F 1024
results/mapping/bsseq.hg19.reverse.clipped.processed.dupmark.bam \
-o results/mapping/bsseq.hg19.reverse.clipped.processed.dupmark.filtered.1.bam

# Generate multi-pileup of reverse and forward strand read mappings:
bin/samtools-0.1.18/samtools mpileup -f data/reference/hg19.fa \
results/mapping/bsseq. hg19.reverse.clipped.processed.dupmark.filtered.2.bam \
results/mapping/bsseq.hg19.forward.clipped.processed.dupmark.filtered.2.bam \
| gzip > results/profile/bsseq.hg19.mpileup.gz

# Call methylation profile from multi-pileup file:
java -jar bin/nxtgen-utils-0.1/NxtGenUtils.jar
ProfileMethylation \
-i $PWD/results/profile/bsseq.hg19.mpileup.gz \
-o $PWD/results/profile/bsseq.hg19.profile \
-r lambda

# Identification of lowly methylated and unmethylated regions (LMR and UMR), using
# methylseekR:
# where ALL_C is a table containing the pooled counts of methylated (M) and unmethylated
# (T) cytosines, in a CpG context, generated by position-wise addition of all the methylation
# profiles from the 38 subjects of the study.
> CPGs <- makeGRangesFromDataFrame(ALL_C, keep.extra.columns=TRUE,
ignore.strand=TRUE, seqnames.field="chr", start.field="pos", end.field="pos")
> library(MethylSeekR)
> library("BSgenome.Hsapiens.UCSC.hg19")
> sLengths=seqlengths(Hsapiens)
> library(rtracklayer)
> session <- browserSession()
> genome(session) <- "hg19"
> query <- ucscTableQuery(session, "cpgIslandExt")
> CpGislands.gr <- track(query)
> genome(CpGislands.gr) <- NA
> CpGislands.gr <- suppressWarnings(resize(CpGislands.grm 5000, fix="center"))
> stat <- calculateFDRs(m=CPGs, CGIs=CpGislands.gr, PMDs=NA, num.cores=1)
> FDR.cutoff <- 5
> m.sel <- 0.5
> n.sel = as.integer(names(stat$FDRs[as.character(m.sel), ][stats$FDRs[as.character(m.sel),
]<FDR.cutoff])[1])
> UMRLMRs <- segmentUMRsLMRs(m=CPGs, meth.cutoff=m.sel, nCpG.cutoff=n.sel,
PMDs=NA, num.cores=1, myGenomeSeq=Hsapiens, seqLengths=sLengths)

# Comparison of DNA methylation patterns between highly and lowly expressed genes
(top1000 and bot1000 genes, as of RNA-Seq data; see Fig2d and FigS8) was conducted
using python & perl scripts available on GitHub at:
https://github.com/zahiaaouabed/correlation-between_methylation_and_expression
```

4.3 RNA-Sequencing

quality trimming and adapter clipping:
fastx_trimer -Q33 -f 10 -z -i [R1.fastq] -o [trim1.R1.fastq.gz]
fastx_trimer -Q33 -f 10 -z -i [R2.fastq] -o [trim1.R2.fastq.gz]
java -jar trimmomatic-0.36.jar PE -phred33 -trimlog trimmomatic.LOG [trim1.R1.fastq.gz] [trim1.R2.fastq.gz] [trim2.R1.fastq.gz] [trim2.R2.fastq.gz] [trim2.U1.fastq.gz] [trim2.U2.fastq.gz] ILLUMINACLIP:adapters_TruSeq.fa:3:30:9 LEADING:5 TRAILING:5 SLIDINGWINDOW:4:15 MINLEN:20
prinseq-lite.pl -fastq [trim2.R1.fastq] -fastq2 [trim2.R2.fastq] -out_good [trimFinal.keep] -out_bad [trim.Final.discard] -trim_tail_left 5 -trim_tail-rigth 5

read alignment with TopHat2:
tophat2 -p 8 --b2-sensitive --no-novel-indels -G [ENSEMBL 75 gtf file] -o [output folder] [hg19 bowtie indices] [R1 fastq] [R2 fastq]

transcript quantification with HTSeq-count:
htseq-count -r name -union -i gene_name --stranded=reverse [name sorted indexed bam] [ENSEMBL 75 gtf file]

differential expression analysis in R with DESeq2:
```
> dds <- DESeqDataSetFromMatrix(countData = countData, colData = colData, design =~ Sex + Age + PMI + pH + RIN + Group)
> dds <- DESeq(dds)
> res <- results (dds)
```

RRHO2 in R:
```
> library(ggplot2)
> library(ggpointdensity)
library(viridis)
> amy_rank <- cbind(data.frame(amy$geneName,stringsAsFactors =TRUE), -log(amy$pvalue, base = 10)*sign(amy$log2FoldChange))
> acc_rank <- cbind(data.frame(acc$Gene.Name,stringsAsFactors =TRUE), -log(acc$pvalue, base = 10)*sign(acc$log2FoldChange))
> amy_rank_overlap <- amy_rank[amy_rank$amy.geneName %in% acc_rank$acc.Gene.Name,]
> acc_rank_overlap <- acc_rank[acc_rank$acc.Gene.Name %in% amy_rank$amy.geneName,]
> amy_rank_overlap_sorted <- amy_rank_overlap[order(amy_rank_overlap$`-log(amy$pvalue, base = 10) * sign(amy$log2FoldChange)`),]
> acc_rank_overlap_sorted <- acc_rank_overlap[order(acc_rank_overlap$`-log(acc$pvalue, base = 10) * sign(acc$log2FoldChange)`),]
> rownames(amy_rank_overlap_sorted) <- amy_rank_overlap_sorted$amy.geneName
> rownames(acc_rank_overlap_sorted) <- acc_rank_overlap_sorted$acc.Gene.Name
> RRHO2(amy_rank_overlap_sorted, acc_rank_overlap_sorted, labels=c("Amygdala","ACC"), plots=TRUE, outputdir="~/OUTPUTDIR/")
```
