# Supplementary material for: Non-CG methylation and multiple histone profiles associate child abuse with immune and small GTPase dysregulation
Source: Nat Commun. 2021 Feb 18;12:1132. doi: 10.1038/s41467-021-21365-3 (PMC7892573; doi:10.1038/s41467-021-21365-3)
Supplement: Supplementary file 3 — Description of Additional Supplementary Files [file 41467_2021_21365_MOESM3_ESM.pdf]

Description of Additional Supplementary Files

Supplementary Data 1: ChIP-Sequencing depth for each sample and each histone mark.

Supplementary Data 2: Genomic sites where significant differences in the number of reads were detected between C and ELA groups, using diffReps, for each histone mark.

Supplementary Data 3: Gene Ontology processes enriched in ChIP-Seq differential sites (DS) identified using GREAT annotation.

Supplementary Data 4: List of genomic sites where a transition in chromatin states (ST) was identified between C and ELA groups, using chromHMM.

Supplementary Data 5: Gene ontology processes enriched in chromatin state transitions (ST), identified using GREAT annotation.

Supplementary Data 6: Gene ontology processes enriched in differentially methylated regions (DMR) in the CG and CAC contexts, identified using GREAT annotation.

Supplementary Data 7: Results of differential expression analysis of RNA-Seq data using DESeq2.

Supplementary Data 8: Gene Ontology enrichment analysis of RNA-Seq data using the ToppGene suite.

Supplementary Data 9: Enrichment analysis of RNA-Seq data using the GSEA algorithm.

Supplementary Data 10: Aggregation of GO terms into main biological pathways affected at transcriptional, DNA methylation, histone modification or chromatin state levels.

Supplementary Data 11: Gene Ontology enrichment analysis of gene lists identified by comparing lateral amygdala and anterior cingulate cortex RNA-Seq data, using RRHO2 (Supplementary Fig.16b-c).
