# Supplementary material for: Non-CG methylation and multiple histone profiles associate child abuse with immune and small GTPase dysregulation
Source: Nat Commun. 2021 Feb 18;12:1132. doi: 10.1038/s41467-021-21365-3 (PMC7892573; doi:10.1038/s41467-021-21365-3)
Supplement: Supplementary file 15 — Reporting Summary [file 41467_2021_21365_MOESM15_ESM.pdf]

nature research

Corresponding author(s): Gustavo Turecki

Last updated by author(s): Oct 17, 2020

Reporting Summary

Nature Research wishes to improve the reproducibility of the work that we publish. This form provides structure for consistency and transparency in reporting. For further information on Nature Research policies, see our Editorial Policies and the Editorial Policy Checklist.

Statistics

For all statistical analyses, confirm that the following items are present in the figure legend, table legend, main text, or Methods section.

n/a	Confirmed	
☐	☒	The exact sample size (*n*) for each experimental group/condition, given as a discrete number and unit of measurement
☐	☒	A statement on whether measurements were taken from distinct samples or whether the same sample was measured repeatedly
☐	☒	The statistical test(s) used AND whether they are one- or two-sided<br>*Only common tests should be described solely by name; describe more complex techniques in the Methods section.*
☐	☒	A description of all covariates tested
☐	☒	A description of any assumptions or corrections, such as tests of normality and adjustment for multiple comparisons
☐	☒	A full description of the statistical parameters including central tendency (e.g. means) or other basic estimates (e.g. regression coefficient) AND variation (e.g. standard deviation) or associated estimates of uncertainty (e.g. confidence intervals)
☐	☒	For null hypothesis testing, the test statistic (e.g. *F*, *t*, *r*) with confidence intervals, effect sizes, degrees of freedom and *P* value noted<br>*Give P values as exact values whenever suitable.*
☒	☐	For Bayesian analysis, information on the choice of priors and Markov chain Monte Carlo settings
☒	☐	For hierarchical and complex designs, identification of the appropriate level for tests and full reporting of outcomes
☐	☒	Estimates of effect sizes (e.g. Cohen's *d*, Pearson's *r*), indicating how they were calculated

Our web collection on statistics for biologists contains articles on many of the points above.

Software and code

Policy information about availability of computer code

Data collection	No software was used for data collection.
Data analysis	Most code and command lines used in this study are detailed in the Supplementary Information File (section 4.). Also, all analytical tools used in this study have already been published and are freely available. These notably include: BSmooth for differential DNA methylation analysis; Kallisto and DESeq2 R packages for gene expression analysis; TopHat v2.1.0 for RNA-Seq alignment; the HTSeq-count v0.6.1p1 for gene quantification; the python scripts region_analysis and deepTools; BWA for alignment of WGBS data; FASTX-Toolkit and Trimmomatic for adapter trimming; Picard for removing ChIP-Seq duplicates; and diffReps for differential enrichment of ChIP-Seq reads.

For manuscripts utilizing custom algorithms or software that are central to the research but not yet described in published literature, software must be made available to editors and reviewers. We strongly encourage code deposition in a community repository (e.g. GitHub). See the Nature Research guidelines for submitting code & software for further information.

Data

Policy information about availability of data

All manuscripts must include a data availability statement. This statement should provide the following information, where applicable:

- Accession codes, unique identifiers, or web links for publicly available datasets
- A list of figures that have associated raw data
- A description of any restrictions on data availability

Raw and processed data reported in this study using brain tissue from the lateral amygdala are publicly available via the Gene Expression Omnibus, with accession GSE151827, at: https://www.ncbi.nlm.nih.gov/geo/query/acc.cgi?acc=GSE151827. Source data are provided with this paper for all figures. RNA-Sequencing data generated using brain tissue from the anterior cingulate cortex is available upon request.

Field-specific reporting

Please select the one below that is the best fit for your research. If you are not sure, read the appropriate sections before making your selection.

- [x] Life sciences
- [ ] Behavioural & social sciences
- [ ] Ecological, evolutionary & environmental sciences

For a reference copy of the document with all sections, see nature.com/documents/nr-reporting-summary-flat.pdf

Life sciences study design

All studies must disclose on these points even when the disclosure is negative.

Sample size	Sample size calculation was not performed. However, we justified experiment sample size based on several previously published reports using similar or even smaller sample sizes (Lutz et al, Am J Psychiatry 2017, 174(12):1185-1194; Labonté et al, Am J Psychiatry 2013, 170(5):511-20; Labonté et al, Arch Gen Psychiatry 2012 69(7):722-31; Sibille et al, Am J Psychiatry 2009 166(9):1011-24) and showing the power to detect significant statistical differences.
Data exclusions	No statistical outlier was removed.
Replication	Validity of our dataset was assessed by conducting systematic comparisons among multiple epigenetic layers, and against gene expression. Each library preparation and sequencing experiment was performed once.
Randomization	Given the nature of our experimental post-mortem design in humans, groups were not randomized. However, they were were balanced for the following covariates: age, post-mortem interval and brain pH. In addition, groups were characterized by the same psychological autopsy methods, therefore avoiding the occurrence of systematic biases.
Blinding	Investigators were blinded to group allocation during data collection and analysis.

Reporting for specific materials, systems and methods

We require information from authors about some types of materials, experimental systems and methods used in many studies. Here, indicate whether each material, system or method listed is relevant to your study. If you are not sure if a list item applies to your research, read the appropriate section before selecting a response.

Materials & experimental systems

n/a	Involved in the study	
☐	☒	Antibodies
☒	☐	Eukaryotic cell lines
☒	☐	Palaeontology and archaeology
☒	☐	Animals and other organisms
☐	☒	Human research participants
☒	☐	Clinical data
☒	☐	Dual use research of concern

Methods

n/a	Involved in the study	
☐	☒	ChIP-seq
☒	☐	Flow cytometry
☒	☐	MRI-based neuroimaging

Antibodies

Antibodies used	H3K4me1: Cell Signaling Technologies, cat #5326BF, lot #2<br>H3K4me3: Cell Signaling Technologies, cat #9751BF, lot#6<br>H3K9me3: Abcam, cat #Ab8898, lot #GR93671-1<br>H3K27me3: Cell Signaling Technologies, cat #9733S, lot #6<br>H3K27ac: Diagenode, cat #pAB-196-050, lot #A1723-0041D<br>H3K36me3: Active motif, cat #MABI0333, lot #12003
Validation	H3K4me1: see https://www.cellsignal.com/products/primary-antibodies/mono-methyl-histone-h3-lys4-d1a9-xp-rabbit-mab/5326<br>H3K4me3: https://www.cellsignal.com/products/primary-antibodies/tri-methyl-histone-h3-lys4-c42d8-rabbit-mab/9751<br>H3K9me3: see https://www.abcam.com/histone-h3-tri-methyl-k9-antibody-chip-grade-ab8898.html<br>H3K27me3: see https://www.cellsignal.com/products/primary-antibodies/tri-methyl-histone-h3-lys27-c36b11-rabbit-mab/9733<br>H3K27ac: see https://www.diagenode.com/en/p/h3k27ac-polyclonal-antibody-premium-50-mg-18-ml?utm_source=CiteAb&utm_medium=listing&utm_campaign=Ab<br>H3K36me3: see https://www.activemotif.com/catalog/details/61021/histone-h3-trimethyl-lys36-antibody-clone-mab-clonemabi-0333

Human research participants

Policy information about studies involving human research participants

Population characteristics	Brain tissue was obtained from the Douglas Bell Canada Brain Bank (DBCBQ; Douglas Mental Health Institute, Verdun, Québec; www.douglasbrainbank.ca). All subjects were Caucasians of French–Canadian descent, a population with a well identified founder effect. Sociodemographic and clinical information are listed in Supplementary Table1. Inclusion criteria for both cases and controls were the following: the subject had to be Caucasian and of French Canadian origin and the subject had to die suddenly without prolonged agonal state. Tissue dissection was performed by histopathologists using reference neuroanatomical maps. Information concerning psychiatric history and socio-demographics was obtained by way of psychological autopsies performed by trained clinicians with the informants best acquainted with the deceased. Diagnoses were obtained using DSM-IV criteria by means of SCID-I interviews adapted for psychological autopsies. Control (C) and early-life adveristy (ELA) groups were matched for age, gender, post mortem interval and RNA integrity values, all meaningful covariates.
Recruitment	The Douglas-Bell Canada Brain Bank (www.douglasbrainbank.ca) collects brain tissue in collaboration with the Montréal coroner office as described in the text. Psychological autopsies were performed by trained clinicians on both controls and cases, with the informants best-acquainted with the deceased, as validated by our group and others. Diagnoses were assigned based on DSM IV criteria. Characterization of early-life histories was based on adapted Childhood Experience of Care and Abuse (CECA) interviews assessing experiences of sexual and physical abuse, psychological abuse, as well as neglect, and for which scores from siblings are highly concordant. We considered as severe early-life adversity reports of non-random major physical and/or sexual abuse during childhood (up to 15 years). Only cases with the maximum severity ratings of 1 and 2 were included. This information was then complemented with medical charts and coroner records. Ethical approval was obtained from the Institutional Review Board of the Douglas Mental Health University Institute. Written informed consent was obtained from the families of each of the deceased subjects prior to inclusion in the study.
Ethics oversight	This study was approved by our IRB (Douglas Mental Health Institute Research Ethics Board), and signed informed consent was obtained from next of kin.

Note that full information on the approval of the study protocol must also be provided in the manuscript.

ChIP-seq

Data deposition

☒ Confirm that both raw and final processed data have been deposited in a public database such as GEO.

☒ Confirm that you have deposited or provided access to graph files (e.g. BED files) for the called peaks.

Data access links<br>*May remain private before publication.*	https://www.ncbi.nlm.nih.gov/geo/query/acc.cgi?acc=GSE151827
Files in database submission	Raw files:<br>S1_BS.bam<br>S2_BS.bam<br>S3_BS.bam<br>S4_BS.bam<br>S5_BS.bam<br>S6_BS.bam<br>S7_BS.bam<br>S8_BS.bam<br>S9_BS.bam<br>S10_BS.bam<br>S11_BS.bam<br>S12_BS.bam<br>S13_BS.bam<br>S14_BS.bam<br>S15_BS.bam<br>S16_BS.bam<br>S17_BS.bam<br>S18_BS.bam<br>S19_BS.bam<br>S20_BS.bam<br>S21_BS.bam<br>S22_BS.bam<br>S23_BS.bam<br>S24_BS.bam<br>S25_BS.bam<br>S26_BS.bam<br>S27_BS.bam

S28_BS.bam
S29_BS.bam
S30_BS.bam
S31_BS.bam
S32_BS.bam
S33_BS.bam
S34_BS.bam
S35_BS.bam
S36_BS.bam
S37_BS.bam
S38_BS.bam
S1_LatAmy_Brain_RNASeq.bam
S2_LatAmy_Brain_RNASeq.bam
S3_LatAmy_Brain_RNASeq.bam
S4_LatAmy_Brain_RNASeq.bam
S5_LatAmy_Brain_RNASeq.bam
S6_LatAmy_Brain_RNASeq.bam
S7_LatAmy_Brain_RNASeq.bam
S8_LatAmy_Brain_RNASeq.bam
S9_LatAmy_Brain_RNASeq.bam
S10_LatAmy_Brain_RNASeq.bam
S11_LatAmy_Brain_RNASeq.bam
S12_LatAmy_Brain_RNASeq.bam
S13_LatAmy_Brain_RNASeq.bam
S14_LatAmy_Brain_RNASeq.bam
S15_LatAmy_Brain_RNASeq.bam
S16_LatAmy_Brain_RNASeq.bam
S17_LatAmy_Brain_RNASeq.bam
S18_LatAmy_Brain_RNASeq.bam
S19_LatAmy_Brain_RNASeq.bam
S20_LatAmy_Brain_RNASeq.bam
S21_LatAmy_Brain_RNASeq.bam
S22_LatAmy_Brain_RNASeq.bam
S23_LatAmy_Brain_RNASeq.bam
S24_LatAmy_Brain_RNASeq.bam
S25_LatAmy_Brain_RNASeq.bam
S26_LatAmy_Brain_RNASeq.bam
S27_LatAmy_Brain_RNASeq.bam
S28_LatAmy_Brain_RNASeq.bam
S29_LatAmy_Brain_RNASeq.bam
S30_LatAmy_Brain_RNASeq.bam
S31_LatAmy_Brain_RNASeq.bam
S32_LatAmy_Brain_RNASeq.bam
S33_LatAmy_Brain_RNASeq.bam
S34_LatAmy_Brain_RNASeq.bam
S35_LatAmy_Brain_RNASeq.bam
S36_LatAmy_Brain_RNASeq.bam
S37_LatAmy_Brain_RNASeq.bam
S38_LatAmy_Brain_RNASeq.bam
S11_13_16_Pool10_LatAmy_Brain_ChIP_H3K27ac_PRODrun.bam
S11_13_16_Pool10_LatAmy_Brain_ChIP_H3K27me3_PRODrun.bam
S11_13_16_Pool10_LatAmy_Brain_ChIP_H3K36me3_PRODrun.bam
S11_13_16_Pool10_LatAmy_Brain_ChIP_H3K4me1_PRODrun.bam
S11_13_16_Pool10_LatAmy_Brain_ChIP_H3K4me3_PRODrun.bam
S11_13_16_Pool10_LatAmy_Brain_ChIP_H3K9me3_PRODrun.bam
S11_13_16_Pool10_LatAmy_Brain_ChIP_Input_PRODrun.bam
S1_25_Pool2_LatAmy_Brain_ChIP_H3K27ac_PRODrun.bam
S1_25_Pool2_LatAmy_Brain_ChIP_H3K27ac_QCrun.bam
S1_25_Pool2_LatAmy_Brain_ChIP_H3K27me3_PRODrun.bam
S1_25_Pool2_LatAmy_Brain_ChIP_H3K27me3_QCrun.bam
S1_25_Pool2_LatAmy_Brain_ChIP_H3K36me3_PRODrun.bam
S1_25_Pool2_LatAmy_Brain_ChIP_H3K36me3_QCrun.bam
S1_25_Pool2_LatAmy_Brain_ChIP_H3K4me1_PRODrun.bam
S1_25_Pool2_LatAmy_Brain_ChIP_H3K4me1_QCrun.bam
S1_25_Pool2_LatAmy_Brain_ChIP_H3K4me3_PRODrun.bam
S1_25_Pool2_LatAmy_Brain_ChIP_H3K4me3_QCrun.bam
S1_25_Pool2_LatAmy_Brain_ChIP_H3K9me3_PRODrun.bam
S1_25_Pool2_LatAmy_Brain_ChIP_H3K9me3_QCrun.bam

S1_25_Pool2_LatAmy_Brain_ChIP_Input_PRODrun.bam
S1_25_Pool2_LatAmy_Brain_ChIP_Input_QCrun.bam
S14_17_19_23_Pool8_LatAmy_Brain_ChIP_H3K27ac_PRODrun.bam
S14_17_19_23_Pool8_LatAmy_Brain_ChIP_H3K27me3_PRODrun.bam
S14_17_19_23_Pool8_LatAmy_Brain_ChIP_H3K36me3_PRODrun.bam
S14_17_19_23_Pool8_LatAmy_Brain_ChIP_H3K4me1_PRODrun.bam
S14_17_19_23_Pool8_LatAmy_Brain_ChIP_H3K4me3_PRODrun.bam
S14_17_19_23_Pool8_LatAmy_Brain_ChIP_H3K9me3_PRODrun.bam
S14_17_19_23_Pool8_LatAmy_Brain_ChIP_Input_PRODrun.bam
S2_10_Pool9_LatAmy_Brain_ChIP_H3K27ac_PRODrun.bam
S2_10_Pool9_LatAmy_Brain_ChIP_H3K27me3_PRODrun.bam
S2_10_Pool9_LatAmy_Brain_ChIP_H3K36me3_PRODrun.bam
S2_10_Pool9_LatAmy_Brain_ChIP_H3K4me1_PRODrun.bam
S2_10_Pool9_LatAmy_Brain_ChIP_H3K4me3_PRODrun.bam
S2_10_Pool9_LatAmy_Brain_ChIP_H3K9me3_PRODrun.bam
S2_10_Pool9_LatAmy_Brain_ChIP_Input_PRODrun.bam
S21_28_33_34_Pool11_LatAmy_Brain_ChIP_H3K36me3_PRODrun.bam
S21_28_33_34_Pool11_LatAmy_Brain_ChIP_H3K27ac_PRODrun.bam
S21_28_33_34_Pool11_LatAmy_Brain_ChIP_H3K27me3_PRODrun.bam
S21_28_33_34_Pool11_LatAmy_Brain_ChIP_H3K4me1_PRODrun.bam
S21_28_33_34_Pool11_LatAmy_Brain_ChIP_H3K4me3_PRODrun.bam
S21_28_33_34_Pool11_LatAmy_Brain_ChIP_H3K9me3_PRODrun.bam
S21_28_33_34_Pool11_LatAmy_Brain_ChIP_Input_PRODrun.bam
S22_29_32_37_Pool5_LatAmy_Brain_ChIP_H3K27ac_PRODrun.bam
S22_29_32_37_Pool5_LatAmy_Brain_ChIP_H3K27ac_QCrun.bam
S22_29_32_37_Pool5_LatAmy_Brain_ChIP_H3K27me3_PRODrun.bam
S22_29_32_37_Pool5_LatAmy_Brain_ChIP_H3K27me3_QCrun.bam
S22_29_32_37_Pool5_LatAmy_Brain_ChIP_H3K36me3_PRODrun.bam
S22_29_32_37_Pool5_LatAmy_Brain_ChIP_H3K36me3_QCrun.bam
S22_29_32_37_Pool5_LatAmy_Brain_ChIP_H3K4me1_PRODrun.bam
S22_29_32_37_Pool5_LatAmy_Brain_ChIP_H3K4me1_QCrun.bam
S22_29_32_37_Pool5_LatAmy_Brain_ChIP_H3K4me3_PRODrun.bam
S22_29_32_37_Pool5_LatAmy_Brain_ChIP_H3K4me3_QCrun.bam
S22_29_32_37_Pool5_LatAmy_Brain_ChIP_H3K9me3_PRODrun.bam
S22_29_32_37_Pool5_LatAmy_Brain_ChIP_H3K9me3_QCrun.bam
S22_29_32_37_Pool5_LatAmy_Brain_ChIP_Input_PRODrun.bam
S22_29_32_37_Pool5_LatAmy_Brain_ChIP_Input_QCrun.bam
S3_12_38_Pool6_LatAmy_Brain_ChIP_H3K27ac_PRODrun.bam
S3_12_38_Pool6_LatAmy_Brain_ChIP_H3K27ac_QCrun.bam
S3_12_38_Pool6_LatAmy_Brain_ChIP_H3K27me3_PRODrun.bam
S3_12_38_Pool6_LatAmy_Brain_ChIP_H3K27me3_QCrun.bam
S3_12_38_Pool6_LatAmy_Brain_ChIP_H3K36me3_PRODrun.bam
S3_12_38_Pool6_LatAmy_Brain_ChIP_H3K36me3_QCrun.bam
S3_12_38_Pool6_LatAmy_Brain_ChIP_H3K4me1_PRODrun.bam
S3_12_38_Pool6_LatAmy_Brain_ChIP_H3K4me1_QCrun.bam
S3_12_38_Pool6_LatAmy_Brain_ChIP_H3K4me3_PRODrun.bam
S3_12_38_Pool6_LatAmy_Brain_ChIP_H3K4me3_QCrun.bam
S3_12_38_Pool6_LatAmy_Brain_ChIP_H3K9me3_PRODrun.bam
S3_12_38_Pool6_LatAmy_Brain_ChIP_H3K9me3_QCrun.bam
S3_12_38_Pool6_LatAmy_Brain_ChIP_Input_PRODrun.bam
S3_12_38_Pool6_LatAmy_Brain_ChIP_Input_QCrun.bam
S4_5_15_20_26_36_Pool1_LatAmy_Brain_ChIP_H3K27ac_PRODrun.bam
S4_5_15_20_26_36_Pool1_LatAmy_Brain_ChIP_H3K27ac_QCrun.bam
S4_5_15_20_26_36_Pool1_LatAmy_Brain_ChIP_H3K27me3_PRODrun.bam
S4_5_15_20_26_36_Pool1_LatAmy_Brain_ChIP_H3K27me3_QCrun.bam
S4_5_15_20_26_36_Pool1_LatAmy_Brain_ChIP_H3K36me3_PRODrun.bam
S4_5_15_20_26_36_Pool1_LatAmy_Brain_ChIP_H3K36me3_QCrun.bam
S4_5_15_20_26_36_Pool1_LatAmy_Brain_ChIP_H3K4me1_PRODrun.bam
S4_5_15_20_26_36_Pool1_LatAmy_Brain_ChIP_H3K4me1_QCrun.bam
S4_5_15_20_26_36_Pool1_LatAmy_Brain_ChIP_H3K4me3_PRODrun.bam
S4_5_15_20_26_36_Pool1_LatAmy_Brain_ChIP_H3K4me3_QCrun.bam
S4_5_15_20_26_36_Pool1_LatAmy_Brain_ChIP_H3K9me3_PRODrun.bam
S4_5_15_20_26_36_Pool1_LatAmy_Brain_ChIP_H3K9me3_QCrun.bam
S4_5_15_20_26_36_Pool1_LatAmy_Brain_ChIP_Input_PRODrun.bam
S4_5_15_20_26_36_Pool1_LatAmy_Brain_ChIP_Input_QCrun.bam
S6_7_Pool4_LatAmy_Brain_ChIP_H3K27ac_PRODrun.bam
S6_7_Pool4_LatAmy_Brain_ChIP_H3K27ac_QCrun.bam
S6_7_Pool4_LatAmy_Brain_ChIP_H3K27me3_PRODrun.bam

S6_7_Pool4_LatAmy_Brain_ChIP_H3K27me3_QCrun.bam
S6_7_Pool4_LatAmy_Brain_ChIP_H3K36me3_PRODrun.bam
S6_7_Pool4_LatAmy_Brain_ChIP_H3K36me3_QCrun.bam
S6_7_Pool4_LatAmy_Brain_ChIP_H3K4me1_PRODrun.bam
S6_7_Pool4_LatAmy_Brain_ChIP_H3K4me1_QCrun.bam
S6_7_Pool4_LatAmy_Brain_ChIP_H3K4me3_PRODrun.bam
S6_7_Pool4_LatAmy_Brain_ChIP_H3K4me3_QCrun.bam
S6_7_Pool4_LatAmy_Brain_ChIP_H3K9me3_PRODrun.bam
S6_7_Pool4_LatAmy_Brain_ChIP_H3K9me3_QCrun.bam
S6_7_Pool4_LatAmy_Brain_ChIP_Input_PRODrun.bam

Processed files:
S1.profile
S2.profile
S3.profile
S4.profile
S5.profile
S6.profile
S7.profile
S8.profile
S9.profile
S10.profile
S11.profile
S12.profile
S13.profile
S14.profile
S15.profile
S16.profile
S17.profile
S18.profile
S19.profile
S20.profile
S21.profile
S22.profile
S23.profile
S24.profile
S25.profile
S26.profile
S27.profile
S28.profile
S29.profile
S30.profile
S31.profile
S32.profile
S33.profile
S34.profile
S35.profile
S36.profile
S37.profile
S38.profile
LatAmy_Brain_RNASeq.raw.counts
S11_13_16_Pool10_H3K27ac.bw
S11_13_16_Pool10_H3K27me3.bw
S11_13_16_Pool10_H3K36me3.bw
S11_13_16_Pool10_H3K4me1.bw
S11_13_16_Pool10_H3K4me3.bw
S11_13_16_Pool10_H3K9me3.bw
S11_13_16_Pool10_Input.bw
S1_25_Pool2_H3K27ac.bw
S1_25_Pool2_H3K27me3.bw
S1_25_Pool2_H3K36me3.bw
S1_25_Pool2_H3K4me1.bw
S1_25_Pool2_H3K4me3.bw
S1_25_Pool2_H3K9me3.bw
S1_25_Pool2_Input.bw
S14_17_19_23_Pool8_H3K27ac.bw
S14_17_19_23_Pool8_H3K27me3.bw
S14_17_19_23_Pool8_H3K36me3.bw

S14_17_19_23_Pool8_H3K4me1.bw
S14_17_19_23_Pool8_H3K4me3.bw
S14_17_19_23_Pool8_H3K9me3.bw
S14_17_19_23_Pool8_Input.bw
S2_10_Pool9_H3K4me3.bw
S2_10_Pool9_H3K27ac.bw
S2_10_Pool9_H3K27me3.bw
S2_10_Pool9_H3K36me3.bw
S2_10_Pool9_H3K4me1.bw
S2_10_Pool9_H3K9me3.bw
S2_10_Pool9_Input.bw
S21_28_33_34_Pool11_H3K27ac.bw
S21_28_33_34_Pool11_H3K27me3.bw
S21_28_33_34_Pool11_H3K36me3.bw
S21_28_33_34_Pool11_H3K4me1.bw
S21_28_33_34_Pool11_H3K4me3.bw
S21_28_33_34_Pool11_H3K9me3.bw
S21_28_33_34_Pool11_Input.bw
S22_29_32_37_Pool5_H3K27ac.bw
S22_29_32_37_Pool5_H3K27me3.bw
S22_29_32_37_Pool5_H3K36me3.bw
S22_29_32_37_Pool5_H3K4me1.bw
S22_29_32_37_Pool5_H3K4me3.bw
S22_29_32_37_Pool5_H3K9me3.bw
S22_29_32_37_Pool5_Input.bw
S3_12_38_Pool6_H3K27ac.bw
S3_12_38_Pool6_H3K27me3.bw
S3_12_38_Pool6_H3K36me3.bw
S3_12_38_Pool6_H3K4me1.bw
S3_12_38_Pool6_H3K4me3.bw
S3_12_38_Pool6_H3K9me3.bw
S3_12_38_Pool6_Input.bw
S4_5_15_20_26_36_Pool1_H3K27ac.bw
S4_5_15_20_26_36_Pool1_H3K27me3.bw
S4_5_15_20_26_36_Pool1_H3K36me3.bw
S4_5_15_20_26_36_Pool1_H3K4me1.bw
S4_5_15_20_26_36_Pool1_H3K4me3.bw
S4_5_15_20_26_36_Pool1_H3K9me3.bw
S4_5_15_20_26_36_Pool1_Input.bw
S6_7_Pool4_H3K27ac.bw
S6_7_Pool4_H3K27me3.bw
S6_7_Pool4_H3K36me3.bw
S6_7_Pool4_H3K4me1.bw
S6_7_Pool4_H3K4me3.bw
S6_7_Pool4_H3K9me3.bw
S6_7_Pool4_Input.bw
S8_30_31_35_Pool7_H3K27ac.bw
S8_30_31_35_Pool7_H3K27me3.bw
S8_30_31_35_Pool7_H3K36me3.bw
S8_30_31_35_Pool7_H3K4me1.bw
S8_30_31_35_Pool7_H3K4me3.bw
S8_30_31_35_Pool7_H3K9me3.bw
S8_30_31_35_Pool7_Input.bw
S9_27_39_Pool3_H3K27ac.bw
S9_27_39_Pool3_H3K27me3.bw
S9_27_39_Pool3_H3K36me3.bw

Genome browser session
(e.g. UCSC)

http://genome.ucsc.edu/cgi-bin/hgTracks?db=hg19&lastVirtModeType=default&lastVirtModeExtraState=&virtModeType=default&virtMode=0&nonVirtPosition=&position=chrX%3A15578261%2D15621068&hgsid=842700757_BMUbvoSamAiEMdbGagOu9lp1XXvm

Methodology

Replicates

No technical replicates were analyzed. Because of the small size of the amygdala lateral nucleus, and the large amounts of tissue required for multiple immune-precipitations and the ChIP-seq analysis of 6 histone marks, tissue from 17 C and 21 ELA subjects were distributed into 7 ELA and 4 C pools, and the 6 marks were analyzed in each of the 11 resulting pools.

Sequencing depth

Sequencing was performed using the Illumina HiSeq2000 to achieve at least 30 and 60 million reads for narrow (H3K27ac, H3K4me3) and broad (H3K27me3, H3K36me3, H3K4me1, H3K9me3) marks, respectively (see Supplementary Figure S1a), following standard

recommandations from the International Human Epigenome Consortium.

Antibodies	H3K4me1: Cell Signaling Technologies, cat #5326BF, lot #2<br>H3K4me3: Cell Signaling Technologies, cat #9751BF, lot#6<br>H3K9me3: Abcam, cat #Ab8898, lot #GR93671-1<br>H3K27me3: Cell Signaling Technologies, cat #9733S, lot #6<br>H3K27ac: Diagenode, cat #pAB-196-050, lot #A1723-0041D<br>H3K36me3: Active motif, cat #MABI0333, lot #12003
Peak calling parameters	No peak calling was conducted. Groups were compared by looking for differential enrichment of ChIP-Seq reads using diffReps, as well as by comparing ChromHMM maps of chromatin states across groups.
Data quality	Coverage profiles were visualized using IGV. Duplicate read removal and GC bias correction were performed with PICARD and deepTools, respectively. Relative and normalized strand cross correlations cutoffs were 0.8 and 1.05, respectively, for narrow marks. ChIP-Seq signal consistency throughout the cohort was assessed through hierarchical clustering of Pearson correlations using deepTools. Identification of differential enrichment sites for each histone mark was done using diffReps with window size 1000bp and sliding step 100bp. A FDR <10% and p <0.0001 for negative binomial test were used as significance cutoffs.
Software	Trimmomatic, BWA, Picard and deepTools were used to pre-process and align the sequencing reads. Global visualization for the ChIP-seq data was done using IGV and ngs.plot. Inter-sample correlations and hierarchical clustering were achieved using deepTools. Identification of differential enrichment sites for each histone mark was done using diffReps. ChromHMM was used to annotate chromatin states.
